# Vaccine-Elicited Tier 2 HIV-1 Neutralizing Antibodies Bind to Quaternary Epitopes Involving Glycan-Deficient Patches Proximal to the CD4 Binding Site

**DOI:** 10.1371/journal.ppat.1004932

**Published:** 2015-05-29

**Authors:** Ema T. Crooks, Tommy Tong, Bimal Chakrabarti, Kristin Narayan, Ivelin S. Georgiev, Sergey Menis, Xiaoxing Huang, Daniel Kulp, Keiko Osawa, Janelle Muranaka, Guillaume Stewart-Jones, Joanne Destefano, Sijy O’Dell, Celia LaBranche, James E. Robinson, David C. Montefiori, Krisha McKee, Sean X. Du, Nicole Doria-Rose, Peter D. Kwong, John R. Mascola, Ping Zhu, William R. Schief, Richard T. Wyatt, Robert G. Whalen, James M. Binley

**Affiliations:** 1 San Diego Biomedical Research Institute, San Diego, California, United States of America; 2 International AIDS Vaccine Initiative (IAVI) Neutralizing Antibody Center at The Scripps Research Institute, Department of Immunology and Microbial Science, La Jolla, California, United States of America; 3 Altravax, Inc., Sunnyvale, California, United States of America; 4 Vaccine Research Center, National Institutes of Health (NIH), Bethesda, Maryland, United States of America; 5 Center for HIV/AIDS Vaccine Immunology and Immunogen Discovery, The Scripps Research Institute, La Jolla, California, United States of America; 6 National Laboratory of Biomacromolecules, Institute of Biophysics, Chinese Academy of Sciences, Chaoyang District, Beijing, China; 7 MRC Human Immunology Unit, Weatherall Institute of Molecular Medicine, University of Oxford, The John Radcliffe Hospital, Oxford, United Kingdom; 8 International AIDS Vaccine Initiative, Design and Development Laboratory, Brooklyn, New York, United States of America; 9 Department of Surgery, Duke University, Duke University Medical Center, Durham, North Carolina, United States of America; 10 Tulane National Primate Research Center, Covington, Louisiana, United States of America; 11 Ragon Institute of MGH, MIT, and Harvard, Cambridge, Massachusetts, United States of America; University of Zurich, SWITZERLAND

## Abstract

Eliciting broad tier 2 neutralizing antibodies (nAbs) is a major goal of HIV-1 vaccine research. Here we investigated the ability of native, membrane-expressed JR-FL Env trimers to elicit nAbs. Unusually potent nAb titers developed in 2 of 8 rabbits immunized with virus-like particles (VLPs) expressing trimers (trimer VLP sera) and in 1 of 20 rabbits immunized with DNA expressing native Env trimer, followed by a protein boost (DNA trimer sera). All 3 sera neutralized via quaternary epitopes and exploited natural gaps in the glycan defenses of the second conserved region of JR-FL gp120. Specifically, trimer VLP sera took advantage of the unusual absence of a glycan at residue 197 (present in 98.7% of Envs). Intriguingly, removing the N197 glycan (with no loss of tier 2 phenotype) rendered 50% or 16.7% (n = 18) of clade B tier 2 isolates sensitive to the two trimer VLP sera, showing broad neutralization via the surface masked by the N197 glycan. Neutralizing sera targeted epitopes that overlap with the CD4 binding site, consistent with the role of the N197 glycan in a putative “glycan fence” that limits access to this region. A bioinformatics analysis suggested shared features of one of the trimer VLP sera and monoclonal antibody PG9, consistent with its trimer-dependency. The neutralizing DNA trimer serum took advantage of the absence of a glycan at residue 230, also proximal to the CD4 binding site and suggesting an epitope similar to that of monoclonal antibody 8ANC195, albeit lacking tier 2 breadth. Taken together, our data show for the first time that strain-specific holes in the glycan fence can allow the development of tier 2 neutralizing antibodies to native spikes. Moreover, cross-neutralization can occur in the absence of protecting glycan. Overall, our observations provide new insights that may inform the future development of a neutralizing antibody vaccine.

## Author Summary

Here we show that native HIV-1 Env spikes expressed in a natural membrane context can induce potent tier 2 nAbs in rabbits. These antibodies reacted exclusively with epitopes present on these trimers and not with isolated Env subunits. Intriguingly, the neutralizing sera were found to take advantage of natural gaps in the carbohydrate defenses of Env spikes of the vaccine strain. Some sera were able to neutralize heterologous isolates, provided that a key, regulating glycan was removed. Overall, these findings suggest that native, membrane-expressed trimers hold promise for further development as vaccine candidates. In the future, by adapting our current findings, we might be able to encourage nAb development to key conserved sites by introducing additional, targeted gaps in the trimer's glycan shell. We suggest that the rare ability to predictably induce potent autologous neutralizing antibodies to field isolates, as we report here, provides a foundation for exploring new strategies aimed at inducing neutralization breadth which is widely expected to be essential for vaccine-induced protection.

## Introduction

Eliciting broadly neutralizing antibodies (bnAbs) is a major goal of HIV-1 vaccine development [1,2]. NAbs block infection by binding to native Env spikes, consisting of trimers of gp120/gp41 heterodimers [2,3]. However, the compact, sequence-diverse, and heavily glycosylated nature of these trimers allows the virus to largely evade neutralization [4,5].

For a neutralizing antibody vaccine to be sufficiently effective, it will have to overcome at least three challenges: i) to consistently induce nAbs in all vaccinees, ii) to induce nAbs that can potently neutralize tier 2 field isolate(s) resembling transmitted strains, and iii) to induce nAbs that are effective against a broad spectrum of tier 2 strains. An ideal vaccine would resolve all these challenges simultaneously. However, most current vaccine candidates usually elicit weak or undetectable autologous tier 2 nAbs, let alone any breadth [1,2,6]. In natural infection, autologous nAbs typically develop within a few months and invariably precede any bnAb development [7]. This may be a reflection of the unprecedented sequence diversity that makes cross-reactive epitopes extremely rare among the exposed targets available on native trimers. A plausible solution may therefore be to first develop a platform that consistently elicits potent autologous tier 2 nAbs, then to use heterologous boosts to try to recapitulate the steps in nAb breadth development in natural infection [8–11]. In other words, we might implicitly solve the challenges described above in a stepwise manner.

Resolving the first challenge (consistent nAb induction) may be facilitated by ensuring that relevant epitope(s) are well-exposed. For example, previous studies have reported that several animals that received JR-FL strain-based immunogens developed modest nAb responses that target the CD4 binding site (CD4bs) [12,13]. To resolve the second challenge (inducing potent tier 2 nAbs), clearly, nAb titers should be sufficient to protect against incident infection. Studies suggest that a ~1:200 nAb ID50 titer (in the TZM-bl assay) can protect against low dose SHIV challenge [14–19]. However, factors such as the nature of the challenge virus, its dose, and nAb specificity complicate any firm estimates. Conservatively, an ID50 titer >1:1,000 might be expected to be protective. In one study, rabbits immunized with a JR-CSF gp120 DNA prime-gp120 protein-boost regimen induced exceptional nAb ID50 titers of >1:10,000 to the tier 2 index virus in the TZM-bl assay, and targeted epitopes involving the gp120 C3/V4 region [20]. Other studies have also shown that DNA prime-soluble protein boost regimens frequently elicit improved nAbs compared to protein-only regimens, although titers usually fall short of what may be protective [13,21,22]. These findings provide reasons to be optimistic that the first two challenges in neutralizing antibody vaccine development can be addressed.

Since functional, trimeric Env spikes stringently resist binding by all but the most precisely targeted nAbs, perhaps only these spikes themselves possess the necessary selectivity to elicit nAbs in a vaccine setting. Indeed, the slow progress in nAb vaccine development may derive from the fact that most Env vaccine candidates insufficiently resemble native spikes [23] and consequently elicit largely “off target” (i.e. non-neutralizing) antibodies [1,2,6]. In an attempt to address this problem, one group generated a "near native" soluble trimer, termed BG505 SOSIP.664, that elicits largely consistent and potent autologous nAbs [24]. This, and any other vaccine approaches based on authentic Env spikes clearly deserve further attention.

To achieve a "near native" conformation, soluble Env vaccine candidates require mutations to increase their stability. However, this comes at a price: to varying extents, these mutations inevitably sacrifice a fully native conformation [23]. In contrast, Env trimers expressed in a natural lipid membrane context do not require trimer-stabilizing mutations, and, unlike their soluble counterparts, fully resemble functional spikes found on infectious virus [25]. Virus-like particles (VLPs) provide one platform for testing membrane-expressed native trimer vaccines. In support of this approach, it is worth noting that all licensed infectious disease vaccines (e.g. HBV, RV, HPV) and many others in development (e.g. influenza, malaria (RTS,S), parvovirus, NDV, RSV, norovirus) are particle-based [26,27]. In the HIV arena, particulate vaccines have so far been explored in the forms of live inactivated viruses, VLPs, liposomes and virosomes (many references are cited in [28]), although none have yet demonstrated a great capacity to elicit tier 2 nAbs.

At least two major factors could underlie the lack of progress in developing nanoparticle vaccines to prevent HIV-1 acquisition. First, germline antibody precursors heavily favor protein-based epitopes over glycan epitopes, as glycans are generally considered to be “self” antigens. Given that accessible protein sites on the trimer are protected by a heavy carbohydrate shell [5,29,30], the germline antibody repertoire may therefore have a limited capacity to engage the trimer, negatively impacting nAb development [8,31–34]. This constraint may be particularly relevant in small animal models whose antibody repertoires may not be well equipped to recognize such challenging antigens [35,36]. By comparison, other, simpler forms of Env such as the gp120 monomer are more accessible and can therefore engage antibody germlines more easily. However, an important drawback is that they lack the ability to selectively elicit nAbs. Despite these challenges, a growing portfolio of broadly neutralizing human monoclonal nAbs (mbnAbs) has revealed various new ways that trimer defenses can be breached and provide paradigms for vaccine design. In many cases, these mAbs target recessed protein-based epitopes that are either bordered by glycans or make direct contacts with glycans [37–49].

A second factor that may underlie the lack of progress in particulate HIV-1 vaccine development may be that their surfaces are contaminated with non-functional forms of Env, including uncleaved (UNC) gp160 and gp41 stumps. These aberrant forms of Env may promote the development of non-neutralizing responses, perhaps at the expense of the development of neutralizing responses directed to the more compact native trimer [28,50,51]. In other words, they may act as antigenic decoys. To address this problem, we previously showed that protease treatment can selectively remove non-functional Env from VLP surfaces, leaving native trimers intact. The resulting particles are termed “trimer VLPs” [25,52]. Strikingly, the IC50 titers of monoclonal antibody (mAb) binding to trimer VLPs and neutralization correlate well [25].

To evaluate the ability of native trimers to induce nAbs, here we immunized rabbits and guinea pigs with high doses of trimer VLPs. Two rabbits developed remarkably potent serum nAbs. We compared these sera to another rare, potent JR-FL neutralizing serum generated in a rabbit immunized with DNA that expresses native trimers followed by a soluble protein boost. All 3 sera targeted quaternary epitopes that took advantage of holes in the trimer’s carbohydrate shell left by the natural absence of glycans in the C2 domain of JR-FL gp120. The VLP sera were also able to neutralize other clade B tier 2 isolates when the same glycan-deficient gap was introduced, suggesting that they target a conserved site to which access is usually regulated by a glycan. We discuss the impetus of these results for the further development of trimer VLP immunogens.

## Results

**Fig 1:**
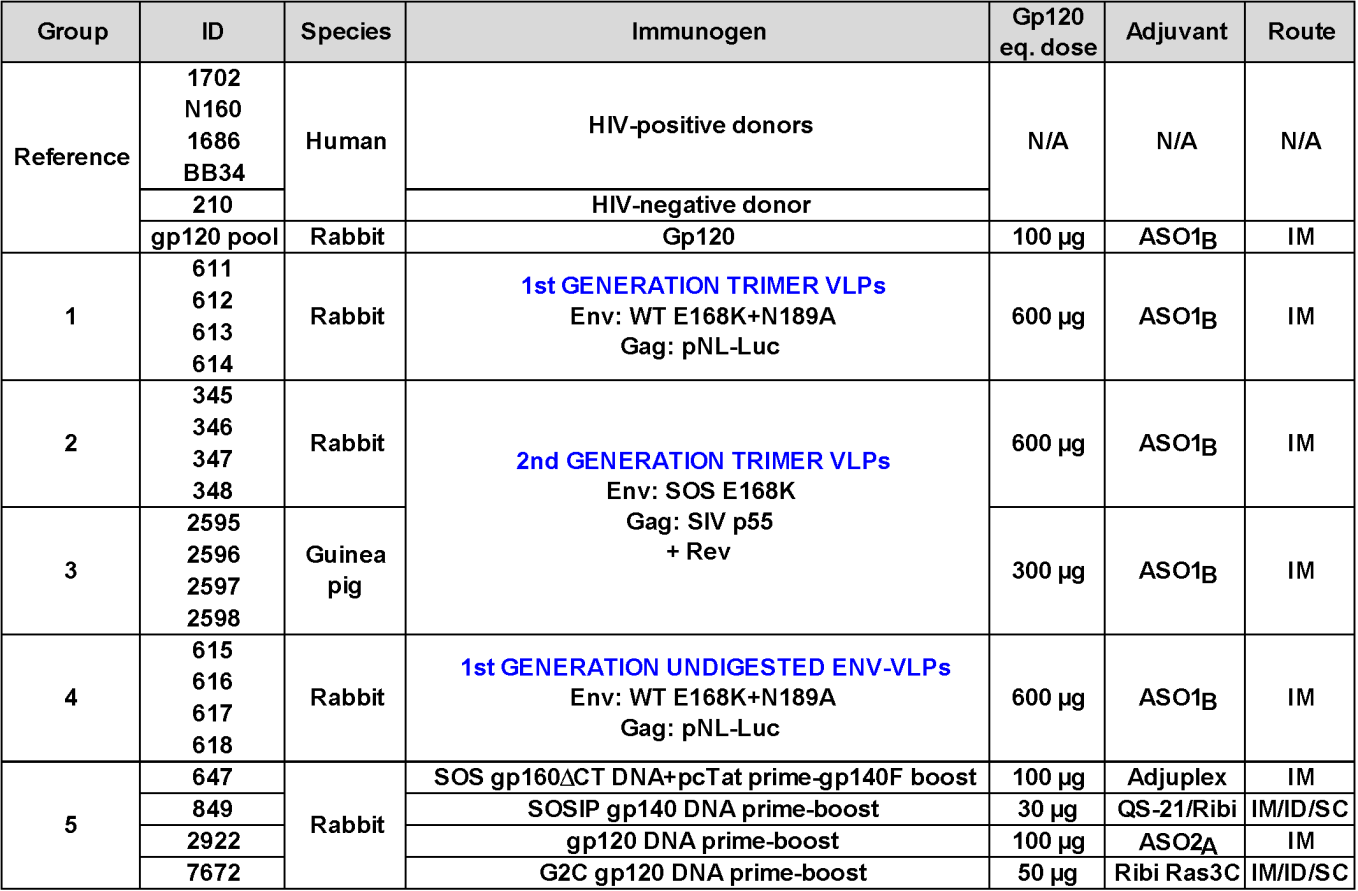
Overview of human plasmas and animal sera. Our plasma and serum panel consisted of human plasmas from 4 HIV-1-infected donors and one uninfected donor, a pool of rabbit sera generated against monomeric JR-FL gp120, groups of rabbit and guinea pig sera generated to various JR-FL Env-VLP-based immunogens. Group 5 animals all received JR-FL-based immunogens via different DNA prime-protein boost protocols. The absolute dose of gp120 and gp140 immunogens is shown. VLP doses are given in estimated gp120 equivalents [28]. Adjuvants and immunization routes are shown (abbreviations are: IM intramuscular, SC subcutaneous and ID intradermal).

Our prior work suggested that the ability of VLPs to induce tier 2 nAbs may be improved by eliminating antigenic interference by non-functional forms of Env [50–52], by increasing the immunogen dose, and by use of a model species with a sufficiently complex antibody repertoire to enable responses to the native Env trimer [28]. Fig 1 provides an overview of a panel of reference sera and five groups of small animal vaccine sera. The reference panel includes four HIV-1 donor plasmas (1702, N160, 1686 and BB34) [53,54], an uninfected human control plasma (210), and an anti-JR-FL gp120 monomer serum pool from rabbits (described previously as "R1" in ref. [28]).

To facilitate comparisons between different vaccine regimens, all animals in groups 1–5 of Fig 1 were immunized with vaccines that present various forms of JR-FL Env. Animals in immunization groups 1–4 were immunized with VLPs bearing gp160∆CT Env in AS01_B_ adjuvant [28,51,53,55]. An E168K mutation, with or without an additional N189A mutation, was used to partially or completely introduce the broad PG9 mAb epitope [25,52,56]. We used higher VLP doses here than in our previous studies [28,50]. Normalized VLP doses used in guinea pigs (375μg/kg; group 3) were higher than those used in rabbits (150μg/kg; groups 1, 2 and 4), assuming mean masses of 0.8kg for guinea pigs and 4kg for rabbits.

VLP immunogens administered to animal groups 1–3 (Fig 1) were treated with proteases to remove non-functional Env, leaving native Env trimers intact (termed “trimer VLPs”) [25,52]. Groups 2 and 3 received VLPs bearing "SOS" mutant Env that introduces a gp120-gp41 disulfide bond [57]. Previous studies have shown that the various Env modifications in these VLP immunogens (i.e. gp41 tail truncation, E168K, N189A and SOS mutations) all have negligible effects on the tier 2 phenotype, compared to the full-length, unmutated JR-FL parent, thereby justifying their use here [53,55,56]. For reference to the trimer VLPs administered to groups 1–3, undigested VLPs were used to immunize group 4 rabbits. During the course of these immunogenicity studies, the plasmids used to express VLPs changed (Fig 1). Thus, 1^st^ generation VLPs, were expressed using the subgenomic pNL-LucR-E- plasmid (abbreviated as pNL-Luc) to induce budding [28]. Conversely, 2^nd^ generation VLPs were expressed using a plasmid expressing SIV p55. In this case, a Rev-expressing plasmid was also co-expressed to enhance Env mRNA export and thereby boost Env expression (Rev is naturally encoded by pNL-LucR-E- used in 1^st^ generation VLPs). In a BN-PAGE analysis, 2^nd^ generation VLPs exhibited markedly improved trimer expression compared to 1^st^ generation VLPs (S1 Fig compare lanes 7 and 8). Like their predecessors, 2^nd^ generation trimer VLPs were also preferentially recognized by neutralizing mAbs [25]. The SOS mutation led to improved trimer expression compared to WT (S1 Fig, compare lanes 5 and 6 to lane 7; [51,53,55]). Protease digestion substantially (albeit incompletely) cleared non-functional Env, including UNC gp160∆CT monomers and gp41 stumps (S1 Fig, compare lanes 1–4 to lanes 5–8; [52]). The residual undigested monomer (see lanes 7 and 8 in S1 Fig) is probably a minor species of UNC gp160 that bears complex glycans, enabling it to survive protease treatments [52]. Overall, this analysis confirms that animals in groups 1–3 were immunized with VLPs bearing predominantly native trimer (S1 Fig, lanes 6 and 8), whereas group 4 animals received VLPs that bear a higher proportion of non-functional Env (S1 Fig, lane 2).

The success of trimer VLPs as immunogens could be adversely affected by protease damage. On the other hand, we know from our previous work that, perhaps surprisingly, VLPs remain fully infectious following protease treatment [25,52]. We also know that trimer VLPs remain intact during ELISA analysis [25,52]. To investigate the stability of our VLP immunogens in more detail, we followed their decay over time at 4°C and 37°C using infectivity and BN-PAGE as readouts of trimer function and stability, respectively (S2 Fig). In brief, we found that trimer VLP infectivity decayed more rapidly (t_1/2_ of 1.4h) than untreated VLPs (t_1/2_ of ~64.5h) at 37°C (S2A Fig). However, residual infectivity (i.e. functional trimer) was nevertheless still detected at 72h. At 4°C full infectivity was retained indefinitely, regardless of protease digestion (S2A Fig). These observations were perfectly complimented by the survival of native trimer under the same conditions, as measured by BN-PAGE-Western blot (S2B Fig). Overall, we conclude that, while trimer VLPs survive protease treatments, they are prone to subsequent decay at physiologic temperatures. Nevertheless, since native trimers and infectivity can still be detected at 72h, they may survive sufficiently long *in vivo* to be able to induce nAbs.

To provide a comparison to our VLP sera, another group of rabbit sera (group 5 in Fig 1) consisted of the best responders from 4 different DNA prime-soluble Env boost immunogenicity studies. The most potent group 5 serum was from animal 647 that had been immunized with pSVIII SOS gp160∆CT plasmid DNA, followed by a single gp140 foldon (gp140F) trimer boost. Nineteen other rabbits immunized with the same or related DNA prime-boost or gp140F only regimens did not develop potent autologous nAbs (S1 Table). Interestingly, the 647 animal was also the only one to develop high titer nAbs against the tier 1A MN strain after 3 DNA primes, i.e., before it was boosted with gp140F trimers (S1 Table). Thus, a particularly effective response to DNA priming may have imprinted tier 2 JR-FL nAbs that were expanded after a single protein boost. Notably, the neutralizing ID50s (particularly against tier 1 viruses) in many animals decreased following the second protein boost (S1 Table). One explanation might be that the rest period between protein boosts 1 and 2 may have been insufficient for B cells to return to a resting state (4 weeks). Another possibility is that there may be competition between lineages initiated by DNA priming and those initiated by the first protein boost. If the protein-initiated responses predominate, this could explain the transient dip in titers at the second boost. Yet another possibility could be an increasing focus on strain-specific epitopes (i.e. JR-FL-specific epitopes not present on the MN or SF162 strains).

The 2922 serum of group 5 was the most potent of 10 rabbit sera arising from a gp120 DNA-soluble gp120 monomer boost vaccination study (S2 Table; [20]). It is worth noting that the autologous nAb ID50s here were weaker than those observed in a previous study using the same regimen based on the JR-CSF strain [20]. This suggests that the Env clone markedly affects nAb induction by this regimen. Serum 849 was derived from an animal immunized with JR-FL SOSIP gp140 in a DNA prime-protein boost regimen [58]. Finally, the 7672 serum resulted from immunization with a mutant gp120 DNA prime-protein boost regimen that contained a graft of the MPER region within the V2 loop (G2C mutant) [59].

### Serum analyses

#### Antigen binding profile

**Fig 2:**
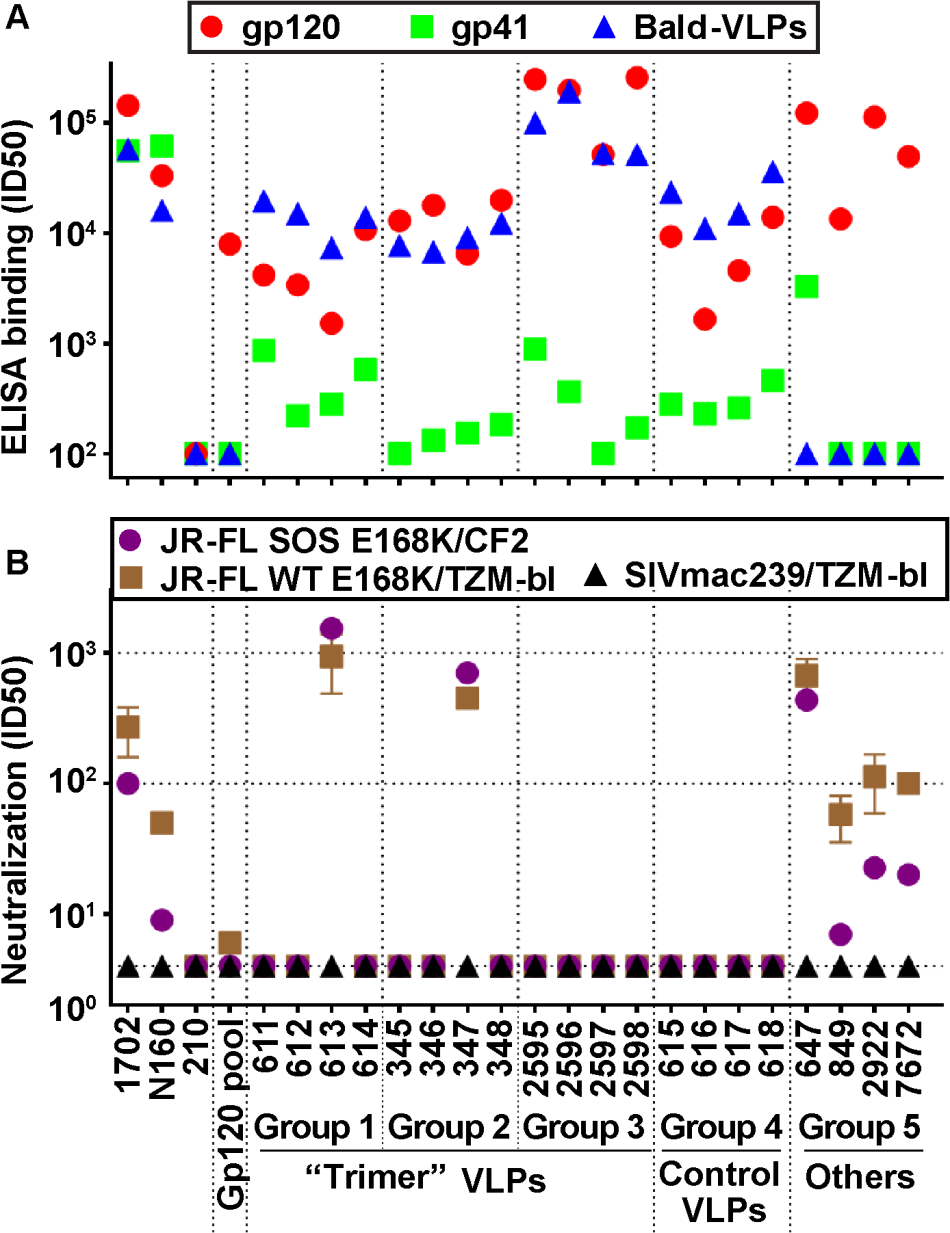
Serum binding and neutralization profiles. A) ELISA titers using recombinant JR-FL gp120, gp41 and bald-VLPs. Data shown are means of at least two repeats. B) Neutralization activity was measured in two different assay formats. TZM-bl cells were used to measure nAbs against the JR-FL WT E168K and SIVmac239 viruses in a "leave in" format. CF2.ThCD4.CCR5 cells were used to measure activity against the JR-FL SOS E168K virus in a “washout” format. All neutralization assays were performed at least three times in duplicate. Mean titers and standard deviations are shown.

Serum binding to monomeric JR-FL gp120, gp41, and bald-VLPs is shown in Fig 2A. HIV-1 donor plasmas 1702 and N160 strongly bound to monomeric gp120, and, as expected, the uninfected control (210) did not (Fig 2A). As shown in S3A Fig, rabbit groups 1, 2 and 4 exhibited modest gp120 titers that were somewhat higher than those in our previous study (group R2-I [28]), perhaps due to the higher immunogen dose. However, this improvement was only significant for group 2 rabbits (*p* = 0.0286, S3A Fig). Three of the 4 group 5 sera exhibited high gp120 titers (Fig 2A), perhaps because soluble forms of Env are more accessible and may therefore more readily elicit antibody responses [60]. Group 3 guinea pig mean gp120 titers were also higher (190,500) than in our previous study (group G2-1; mean titer = 19,500; [28]), again perhaps due to the higher immunogen dose.

HIV-1-infected donor plasmas 1702 and N160 both reacted strongly with recombinant gp41 (Fig 2A). The 647 serum (group 5) also had a high gp41 titer, probably because immunodominant gp41 epitopes are exposed on gp140 foldon. As expected, the uninfected human plasma control (210) and all the gp120-based immune sera (gp120 pool, 2922 and 7672) did not bind to gp41, nor did group 5 serum 849, because SOS mutations eliminate the immunodominant gp41 cluster I and II epitopes, as we reported previously [60]. VLP sera exhibited lower gp41 titers than in our previous study (depicted as group R2-1 in S3B Fig; [28]). In some cases (groups 1 and 4), this may be due to the reduced proportion of non-functional Env present on VLPs bearing E168K and N189A mutations (S1 Fig, compare lane 1 and 2 [25,28]). The low titers in groups 2 and 3 can be explained by the fact that the SOS mutation eliminates major gp41 immunodominant epitopes, as mentioned above (*p* = 0.0286; Figs 2 and S3B).

All 16 VLP sera (groups 1–4) bound effectively to bald-VLPs bearing no surface Env (Fig 2A). High ID50 titers were also observed in HIV-1-infected donor plasmas, but, as expected, not in the uninfected control. These findings may at least in part be explained by lipid-reactive antibodies [28] that are benign and ubiquitous in nature [61]. Indeed, none of our VLP-immunized animals exhibited any ill effects or toxicity related to immunizations. Like gp120 titers, the bald-VLP titers in our VLP sera were moderately higher than in our previous study (S3C Fig; [28]). This difference was only significant, however, for group 4 (*p* = 0.0286; S3C Fig). Bald-VLP titers in group 3 guinea pigs (mean 99,852) were also higher than those in G2-I guinea pigs (mean 13,333) of our previous study (Fig 3 in [28]). As expected, group 5 sera and the rabbit gp120 serum pool exhibited no bald-VLP reactivity.

**Fig 3:**
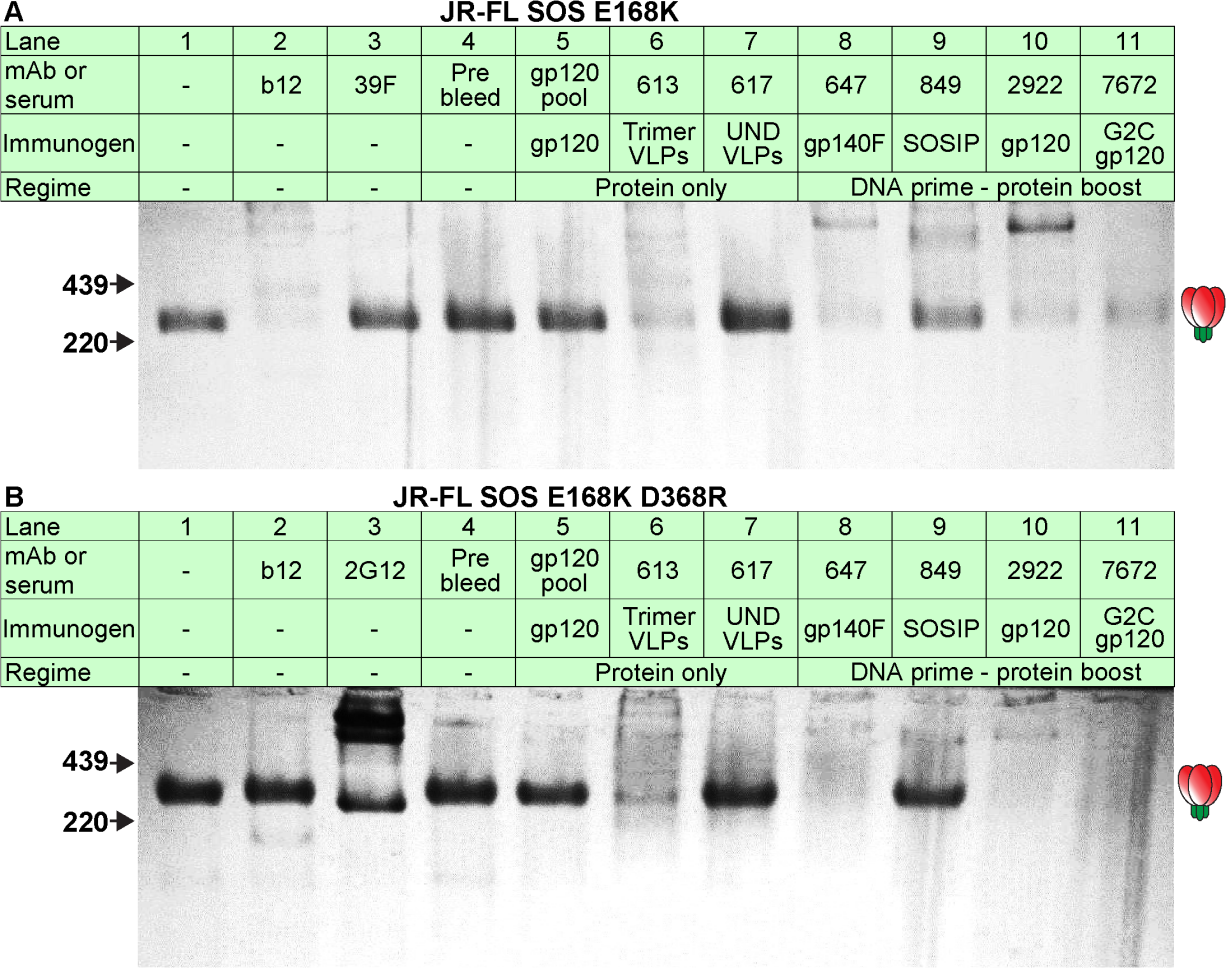
Serum recognition of the native trimer and effects of a key mutation in the CD4 binding loop. MAbs and serum binding to the native Env trimer was assessed in BN-PAGE shift assays. Briefly, mAbs (30μg/ml) or sera (1:2 dilution) were incubated with protease-digested 2nd generation A) JR-FL SOS E168K VLPs or B) JR-FL SOS E168K+D368R VLPs. The VLPs were then washed and lysed, and Env was resolved by BN-PAGE-Western blot. Ferritin was used as a molecular weight marker. The unliganded trimer is indicated by a cartoon. The abbreviation “UND VLP” (immunogen for rabbit 617) refers to “undigested VLPs”.

#### Neutralization

To provide a snapshot of autologous JR-FL neutralization in different assay scenarios, we measured serum activities against the SOS E168K and WT E168K viruses using CF2 and TZM-bl as target cells, respectively (Fig 2B), as in our recent study [28]. We previously showed that WT and SOS mutant viruses exhibit comparable neutralization sensitivities [28,53]. The TZM-bl assay is generally more sensitive than the CF2 assay, largely because antibodies are left incubating with the virus and cells for the duration of the assay, rather than being washed out after the initial infection period [28].

HIV-1-infected donor plasmas 1702 and N160 exhibited modest ID50s titers that were higher in the TZM-bl assay (Fig 2B). “Trimer VLP” sera 613 and 347 of groups 1 and 2, respectively, exhibited remarkably high ID50s in both assays—more than an order of magnitude more potent than any of the other VLP sera here or in our previous VLP immunogenicity studies [28,50]. In multiple repeat assays in various formats, the 613 and 347 sera consistently neutralized the JR-FL strain with mean titers of 1:915 and 1:622, respectively. Purified IgG from the 613 serum also exhibited neutralizing activity comparable to its parent serum, confirming that neutralization was IgG-mediated. The use of a WT versus SOS background, E168K or E168K+N189A mutations, CF2 versus TZM-bl target cells, and “wash out” versus “leave in” formats all had only minor effects on the neutralization titers of these sera. Notably, the serum from animal 347 was collected after only 3 immunizations. This animal died unexpectedly shortly before it was scheduled for its fourth immunization. The nAb titer of serum 613 increased >10 fold between bleeds 3 and 4, suggesting that at least 4 shots may be necessary for nAb titers to mature.

Group 5 serum 647 showed similarly remarkable ID50s in both assays (Fig 2B), consistent with the data in S1 Table. The other 3 sera of group 5 exhibited ID50s of ~1:100 in the TZM-bl assay and weaker activity in the CF2 washout assay, also consistent with earlier data ([58,59]; S2 Table). These titers are still far higher than typical vaccine titers that usually range from <4 to ~1:30, as exemplified by the gp120 serum pool, the other VLP sera in Fig 2B, and most of the sera analyzed in S1 and S2 Tables (note that the data in S2 Table used an 18h virus-serum incubation step that tends to lead to higher ID50 titers). None of the sera detectably neutralized other tier 2 strains—a point we will return to below. They also failed to neutralize the SIV control, confirming that the observed JR-FL neutralizing activity is specific.

Representative neutralization curves of vaccine sera against the tier 2 parent strain are shown in S4 Fig All three potent sera (from animals 613, 347 and 647) mediated dose-dependent neutralization of WT and SOS viruses in the CF2 and TZM-bl assays. At low dilutions, this neutralizing activity approached 100%. Similar ID50s were observed in 3 independent laboratories (RW, DM and JB). In contrast, two non-neutralizing sera (2595 and 617) exhibited little or no neutralizing activity (S4 Fig).

We next examined tier 1 nAb titers. Previously, we reported that A328G mutant JR-FL Env is “globally” neutralization sensitive (see S5 Fig in [28]), probably due to a partial unraveling of the trimer [62,63]. This mutant virus can therefore detect "off target" tier 1 nAbs that match the JR-FL vaccine strain. In S3 Table, we measured tier 1 nAbs against this JR-FL A328G mutant and the heterologous SF162 tier 1 isolate. Groups 1 and 2 exhibited weaker tier 1 nAb titers compared to group 4 (S3 Table), although these differences were not statistically significant (*p* = 0.7845; S5 Fig). Consistent with its weak gp120 titer (Fig 2A), animal 616 of group 4 exhibited undetectable activity in this assay, suggesting that it was a poor vaccine responder.

The guinea pigs of group 3 developed significantly higher tier 1 nAb titers than the rabbits in the current study and the guinea pigs in our previous study (*p*<0.005; S5 Fig). This reflects their relatively high gp120 binding titers (Fig 2A). These ID50s were similar to those of group 5, generated in response to various DNA prime-protein boost regimens (S3 Table). It is possible that VLPs are broken down more rapidly in guinea pigs, promoting the development of tier 1 nAbs that recognize degraded forms of Env. Conversely, the weaker tier 1 nAbs in trimer VLP-immunized rabbits suggest that this degradation process may be slower in this species. The presence of residual non-native Env on trimer VLP surfaces (S1 Fig) could also account for the weak tier 1 responses in these animals.

Since all 3 potent vaccine sera exhibited low tier 1 ID50s (S3 Table), we investigated a possible relationship, if any, between vaccine-elicited tier 1 and 2 nAb titers (S6 Fig). This revealed a poor inverse correlation (*r* ^2^ = 0.05942) that was not significant (*p* = 0.287), suggesting that different nAbs account for the tier 1 and tier 2 responses and that these responses develop independently.

NAbs to the more resistant tier 1 SF162 isolate (S3 Table) were low or undetectable in all VLP sera, except for guinea pig serum 2595. Group 5 serum 647 also exhibited a very low titer (S1 and S3 Tables), but titers in the 2922 and 7672 sera were somewhat higher (S2 and S3 Tables). Notably, the SF162 titer of serum 2922 in S2 Table was ~80-fold higher compared to that in S3 Table. This is probably because prolonged virus-serum incubation (as in used in data in the S3 Table) enhances assay sensitivity.

### Potently neutralizing sera recognize native trimers in a D368-independent manner

We previously showed that neutralization exhibits an excellent correlation with the ability of antibodies to recognize native trimers as observed in BN-PAGE-Western blot [3,25,50–54,64,65]. Consistent with their neutralizing activities, mAb b12 and neutralizing sera from animals 613, 647, 849, 2922 and 7672 all bound to the native SOS E168K trimer, as evidenced by depletion of the unliganded trimer (Fig 3A, lanes 2, 6 and 8–11). Trimer binding by serum 849 was rather weak, consistent with its modest neutralizing ID50 (Fig 2B). All other samples failed to bind parent JR-FL trimers, consistent with their lack of neutralizing activity (Fig 3A). Recognition of SOS D368R mutant trimers by the 613, 647, 2922 and 7672 sera suggest that these sera recognize native trimers via D368-independent epitopes. In similar studies, serum 347 also recognized native trimers in a D368-independent manner. In contrast, binding by mAb b12 and serum 849 (SOSIP) was eliminated, consistent with their dependency on residue D368 for trimer binding [12]. It is worth noting here that IgG-trimer complexes are rarely visible in these experiments, as we reported previously [25]. This is likely to be due to IgG bivalency and flexibility. Thus, a single IgG can potentially engage two trimers, two or three individual IgGs can engage a single trimer and higher order IgG-trimer complexes are possible. This plethora of possibilities probably explains the general lack of well-defined trimer-IgG complexes in these experiments. In fact, several products are often observed, reflecting the various complex combinations. This issue may be exacerbated by IgG flexibility, which may cause band smearing.

To detect any MPER nAbs, we evaluated sera in a post-CD4CCR5 neutralization assay format we described previously [55], and observed no activity (S4 Table). In contrast, the previously reported BB34 serum (serving here as a reference control) showed some activity [54]. To test whether these sera share contacts on Env with known broadly neutralizing mAbs, we also examined the effects of various knockout mutants on neutralization sensitivity, including N160A, N295Q, and N332Q. None of these mutations eliminated the neutralizing activities of the 613, 347 and 647 sera, suggesting that they must contact the trimer via other sites (S4 Table).

### Potently neutralizing sera recognize quaternary epitopes

The poor tier 1 neutralizing activities of our potent vaccine sera (S3 Table) raise the possibility that nAbs may target quaternary tier 2 epitope(s) that are inaccessible on more sensitive HIV-1 strains. To address this possibility, we tested the ability of JR-FL monomeric gp120 and gp140F trimer to interfere with their neutralizing activities (S7 Fig). A D368R mutation was introduced into these soluble Env competitors to prevent them from binding to cellular CD4 and thereby directly inhibiting infection. We already showed that several of our sera (613, 347, 647, 2922, and 7672) recognize native Env trimers independently of the D368R mutation (Fig 3). Therefore, any interference would indicate that nAb epitopes are present on these soluble forms of Env. In S7 Fig, gp120 monomer and gp140F trimer both interfered with mAb 2G12 neutralization, but not with b12 neutralization. The latter was expected, because the D368R mutation is known to ablate b12 recognition. The soluble Envs also interfered with neutralization by group 5 serum 2922, as expected, considering this animal received a gp120 DNA prime, gp120 monomer boost regimen. In contrast, the sera from animals 613, 347 or 647 were unaffected (S7A and S7B Fig), suggesting that their neutralizing epitopes are not expressed on soluble forms of Env. This contrasts with the soluble Env interference we observed with VLP sera in our previous study [28].

Given that gp140F trimers were used as a protein boost in animal 647, the lack of D368R gp140F trimer interference was rather surprising. However, since quaternary nAbs developed after only one protein boost (S1 Table), then the boosting may have merely expanded antibodies that had been imprinted by DNA priming. The exceptional MN neutralizing activity present in this animal before protein boosting (S1 Table) suggests an unusually strong response to DNA priming in this animal that could be consistent with such imprinting. Nevertheless, the tier 1 nAbs against the JR-FL A328G were susceptible to monomeric gp120 interference, suggesting they are mediated by antibodies that do not depend on quaternary epitopes, unlike the tier 2 nAbs in this serum (S7C Fig).

**Fig 4:**
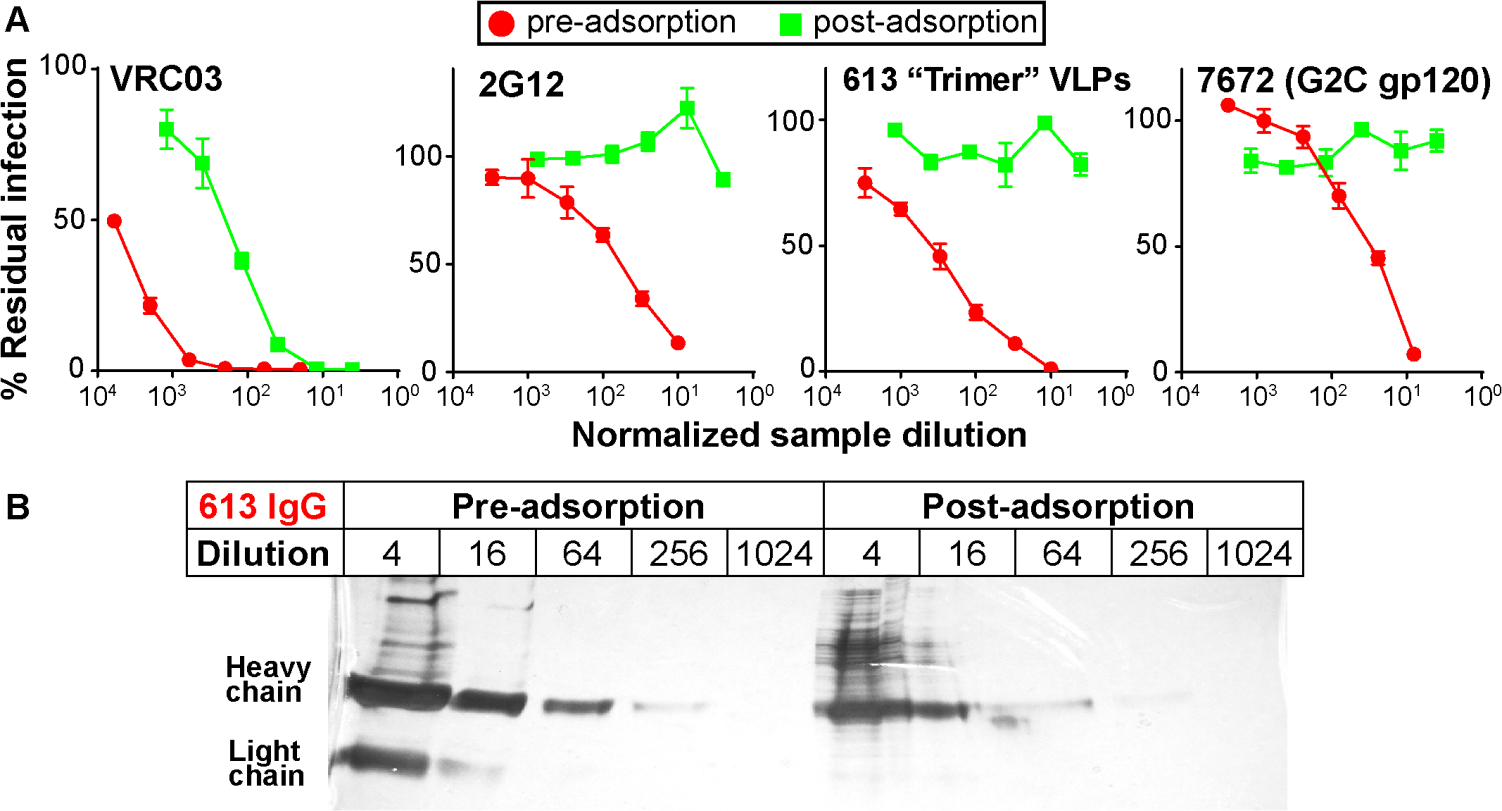
Adsorption to native Env trimer expressed on cell surfaces removes potent serum neutralizing activities. IgG was first protein A-purified from sera and adjusted to the original serum concentration (~10mg/ml). Samples were then adsorbed repeatedly to native Env trimer-expressing cells. IgG was then re-isolated and adjusted to the original volume. A) Pre- and post-adsorption IgG samples were then assessed for neutralizing activity against JR-FL gp160∆CT WT E168K in the TZM-bl assay. B) Purified IgG from serum 613 was monitored before and after adsorption by silver stain. Each sample was titrated.

We next assessed the ability of repeated serum adsorption to densely packed cells expressing native Env trimers to deplete neutralizing activity [44]. Prior to adsorption, pure IgG was extracted from the 613 and 7672 sera (the latter was included as a control). IgG concentrations were then adjusted to match that of their respective parent sera (as verified by ELISA). After adsorption to cells, IgG was again purified and then adjusted back to its pre-adsorption volume. This process resulted in heavily depleted VRC03 and 2G12 neutralization (30- to 100-fold reduction; Fig 4A). NAbs in the 613 and 7672 sera were also dramatically depleted (>100 fold). However, the 613 serum IgG was only modestly depleted by adsorption (~4-fold; Fig 4B). Therefore, the loss in neutralizing activity was due to specific adsorption to the native trimer, rather than non-specific antibody loss during the adsorption process. This data supports the idea that the 613 serum contains nAbs directed to quaternary epitope(s).

### Potent sera target epitopes that overlap the CD4 binding site

**Fig 5:**
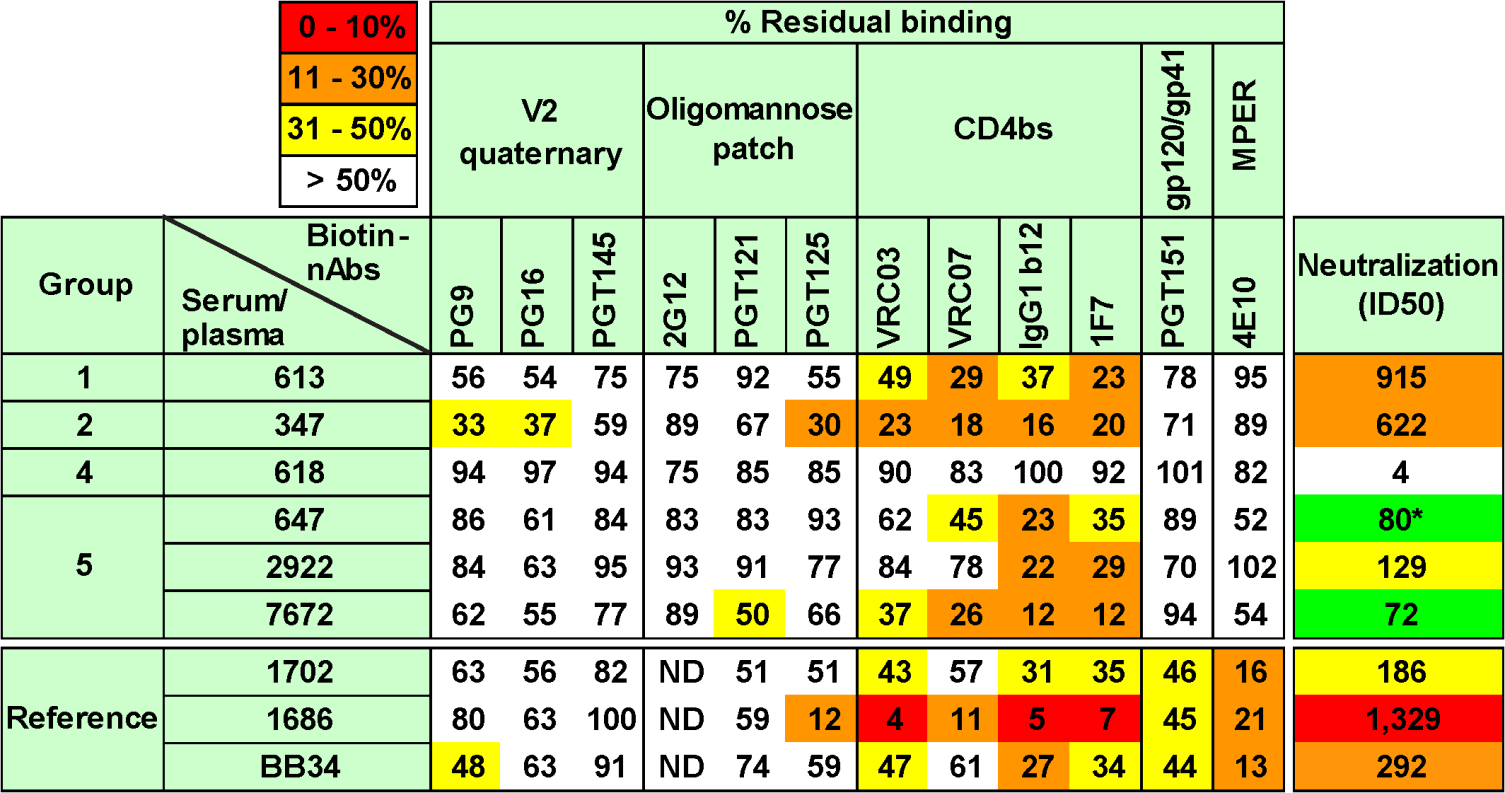
Potent vaccine sera target epitopes that overlap the CD4 binding site. Selected vaccine sera (1:10 dilution) were competed against various biotinylated monoclonal bnAbs for binding to SOS E168K trimer VLPs by ELISA. E168K+N189A mutant trimer VLPs were used for assays involving biotinylated V2 quaternary mAbs to ensure a full epitope knock in [56]. Three previously mapped HIV-1-infected plasmas were included for reference purposes [54]. These are (epitopes in parentheses) 1702 (some MPER and unmapped nAb), 1686 (predominantly CD4bs nAb) and BB34 (predominantly MPER nAb). Numbers are expressed as the residual % binding titer of biotinylated mAb in the presence of competitor compared to its titer in the presence of a control rabbit prebleed or HIV-1 seronegative human plasma (from donor 210). Each assay was performed at least in duplicate. Standard errors are shown in S8 Fig.

To further map the potent vaccine sera, we assessed their ability to compete with various mAbs for binding to trimer VLPs [28,60]. Here, competitions reflect direct epitope overlaps or conformational relationships on the native Env trimer. For simplicity, we infer them to indicate epitope overlaps. In addition to our vaccine sera, for reference purposes, three previously mapped HIV-1 plasmas were also analyzed ([54]; see Fig 1). All rabbit serum-mAb competitions were referenced to a rabbit prebleed control. Similarly, HIV-1 donor sera were referenced to the HIV-1 seronegative human plasma from donor 210. Our panel of biotinylated mAbs is organized horizontally in Fig 5, starting with those directed to epitopes at the membrane-distal apex of the trimer on the left and ending on the right with those directed to the membrane-proximal ectodomain region (MPER). Competitions were considered as significant when biontinylated mAb binding was reduced to <50% of control levels (indicated by colored squares). A non-neutralizing reference serum from rabbit 618 did not compete with any mAb to <50%, suggesting that this cutoff is sufficiently stringent to rule out any non-specific inhibition by our vaccine sera. Multiple representatives of major epitope clusters were assayed to ensure the consistency of any inhibitions within these clusters. Each assay was repeated at least 2 times. The generally low standard errors we observed in S8 Fig reveal the consistent patterns in these repeats. In S9A Fig, we show that each mAb inhibited its biotin-labeled counterpart and other mAbs within each epitope cluster to <10% of control levels (diagonal boxes from top left to bottom right). In representative inter-cluster competitions shown in S9A Fig, as expected, 2G12 was unable to inhibit CD4bs nAb binding. Similarly, various gp120-specific nAbs were unable to inhibit the binding of biotinylated 4E10. However, a competitive inter-cluster relationship between PGT125 and CD4bs nAbs was observed, consistent with the known overlap between these epitopes [42,66]. As with the serum-mAb competitions (S8 Fig), the standard error of repeated mAb-mAb competitions (all assays were performed at least twice) were generally low. Representative titrations of biotinylated mAbs in the presence or absence of unlabeled mAb competitors are shown in S10 Fig and exemplify the strong mAb-mAb self-competition in S9 Fig.

Of all the polyclonal samples, the potently neutralizing HIV-1-infected donor plasma 1686 showed the highest overall competition, strongly inhibiting binding by all 4 CD4bs mAbs (Fig 5). This agrees with our previous mapping of this sample using other methods [54]. The 1686 plasma also competed with mAb PGT125 (Fig 5), consistent with the PGT125-CD4bs overlap mentioned above (S9 Fig). Competition of most other mAbs we tested was marginal, except for 4E10. In S10 Fig, representative titrations show strong plasma 1686 competition of mAb VRC03 directed to the CD4bs, but little or no competition of mAb PG9. The other 2 more weakly neutralizing human plasmas (BB34 and 1702) showed modest CD4bs competition, as well as 4E10 competition, consistent with their known MPER nAb activities ([54]; S4 Table). The marginal (but consistent) competition of VRC03 and to some extent with PG9 by these two plasmas is shown in S10 Fig. Overall, these findings confirm the specificity and reproducibility of this competition assay.

All the neutralizing rabbit sera showed significant competition with CD4bs mAbs VRC03, VRC07, b12 and 1F7 (Figs 5 and S10). Of these, the 347 serum exhibited the strongest activity and, like the human 1686 plasma also competed moderately with mAb PGT125. This serum also competed modestly, but consistently with mAbs PG9 and PG16 (Figs 5 and S10). We suggest that serum 347 binding to trimer has an allosteric effect on the integrity of these V2/quaternary epitopes. The vaccine sera showed little competition with other mAbs. The 613 and 7672 sera showed a similar pattern, competing with all 4 CD4bs nAbs, with possible weak inhibition of PGT125, PG9 and PG16. The 647 and 2922 sera showed weaker competition that was also focused on the CD4bs. In the case of the 647 serum, the unexpectedly weak competition was because limited sample availability meant that we had to use serum from a bleed taken 4 weeks after the immune bleed used elsewhere in this study, by which time the neutralizing ID50 had fallen to ~1:80.

The patterns of inhibition by vaccine sera are represented as raw titrations in S10 Fig, where VRC03 binding is clearly perturbed, but the effects on PG9 and 2G12 are weak or absent, respectively. Although the extent of competition by vaccine sera was in no case as complete as with mAb-mAb competitions (S10 Fig), competition patterns were consistent between assays (S8 Fig). Furthermore, in most cases, inhibition of all four different mAbs within the CD4bs cluster was detected (Fig 5). The competition profiles of our vaccine sera patterns are also strongly supported by our observing expected patterns of competition by HIV-1 donor sera. It may be no coincidence that all vaccine sera overlap with the CD4bs, considering that this is one of the few partially exposed protein patches on the otherwise densely glycan-populated trimer. The observation that the 347 and 7672 sera exhibited stronger competition of this site than the 613 serum, despite having somewhat lower neutralizing ID50s may indicate that their neutralizing activities have a greater overlap with the CD4bs.

### Potent vaccine-elicited nAbs depend on the gp120 C2 region

**Fig 6:**
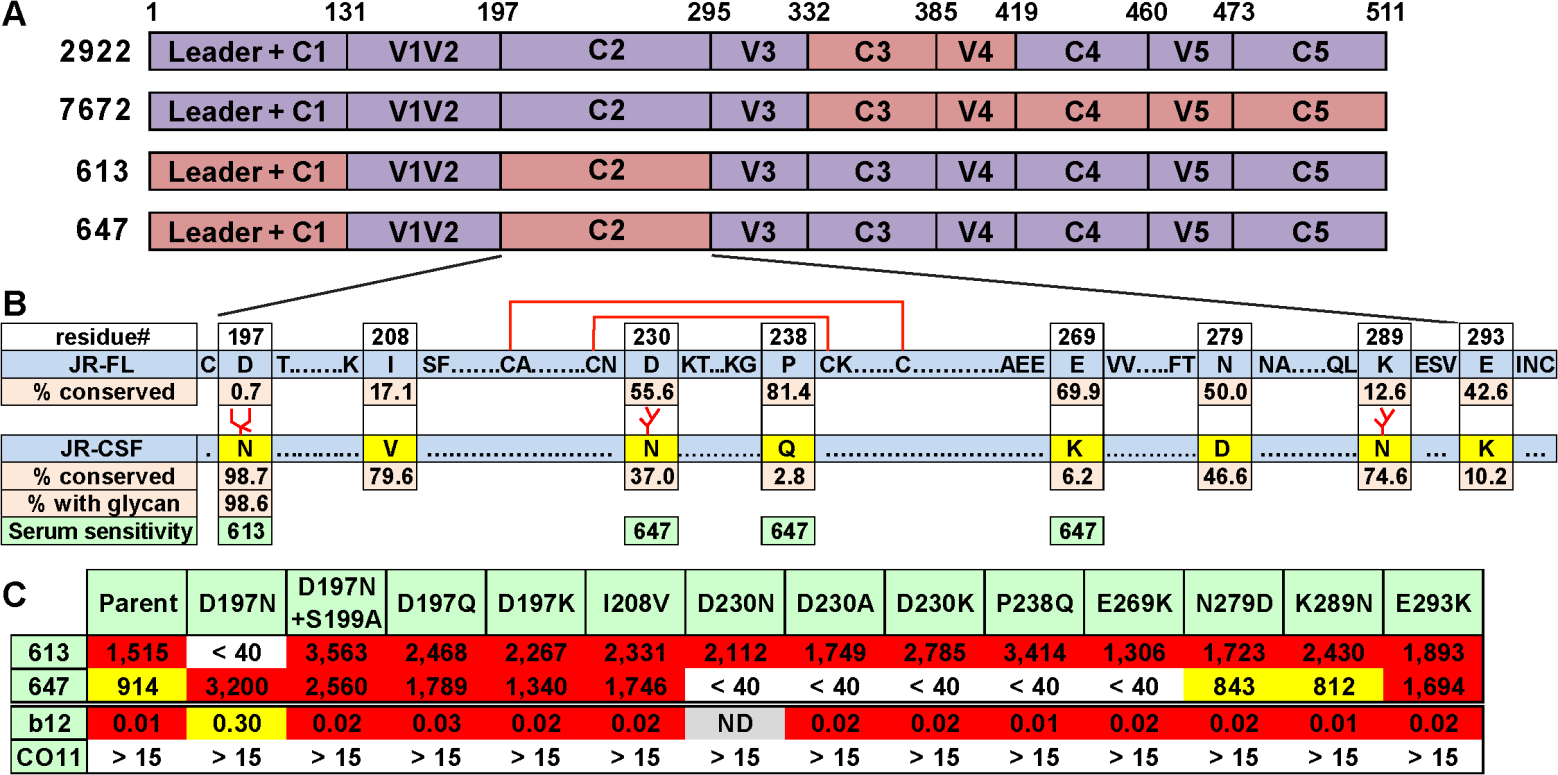
Domain and residue-level mapping of potent rabbit sera. A) Domains that determine the neutralizing activities of each of the 4 sera shown were inferred from the chimera analysis in S11 Fig. Domains are color-coded: purple indicates JR-FL Env domains, salmon indicates JR-CSF Env domains. B) C2 domains of JR-FL and JR-CSF Envs were aligned. To save space, some conserved residues are abbreviated as dots. Eight differences in the JR-CSF sequence are shown in yellow. The conservation of JR-CSF residues at each of these positions is shown. A disulfide-bonded hairpin is shown by red lines. The sensitivities of the 613 and 647 sera to JR-CSF mutations are shown in green, C) 613 and 647 serum neutralization ID50s of JR-FL clones bearing JR-CSF-based residue substitutions. Each mutant was also evaluated for neutralization sensitivity (IC50s in μg/ml) to mAbs b12 and CO11.

Like all other vaccine sera described to date, none of our sera significantly neutralized tier 2 viruses, aside from the autologous JR-FL parent virus. The lack of JR-CSF cross-neutralization is sobering, given that this strain was isolated from the same donor as JR-FL and therefore exhibits a degree of sequence homology. This lack of JR-CSF cross-neutralization, however, has the benefit of allowing us to map our sera by measuring their activities against a series of JR-FL Env-based chimeric pseudoviruses with various JR-CSF Env domain swaps [20,67]. Most of these chimeras were infectious (S11A Fig). All of the functional clones resisted neutralization by the V3 loop-specific mAb CO11 [28], suggesting that they retain a tier 2 phenotype. The potent sera from animals 613 and 647 neutralized all of the functional chimeras (S11A Fig). This suggests that they target epitopes involving the JR-FL C1 and/or C2 regions that were not represented in the chimera neutralization analysis, as they were not sufficiently functional (S11A Fig). In contrast, sera 2922 and 7672 targeted epitopes affected by the C-terminal portion of gp120 (S11A Fig). Specifically, the 2922 gp120 DNA prime-protein boost sera targeted the C3V4 region, which is similar to the epitope targets of the sera previously reported using this regimen using the JR-CSF strain [20], suggesting a common nAb development pathway. The 7672 G2C gp120 prime-boost serum was knocked out by a C3-C5 chimera (clone 8070), although its activity could not be further resolved by less substantial domain swaps (S11A Fig). These results are summarized in Fig 6A.

Since the competition data in Fig 5 suggested that our potent sera target CD4bs-like epitopes, we first investigated the possible role of the C2 region in the 613 and 647 epitopes, due to its close proximity to the CD4bs. Alignment of JR-FL and JR-CSF C2 regions (Fig 6B) reveals 8 amino acid differences, including 3 additional glycan sequons in JR-CSF. These substantial differences could account for the lack of infectivity of the C2 domain swap mutants (8076 and 8086) in S11A Fig—JR-FL trimers may be unable to accommodate all 3 extra glycans. An analysis of JR-FL-based point mutants at the 8 variant sites revealed that the 613 serum was sensitive to a D197N mutation at the base of the V1V2 loop that introduces one of the three additional JR-CSF glycans (Fig 6B and 6C). Several other D197 mutants (D197N+S199A, D197Q and D197K) that do not introduce a new glycan had no effect, suggesting that the N197 glycan regulates 613 serum neutralization (Fig 6C). None of the other C2 mutants affected neutralization by the 613 serum. It is worth noting that the introduction of the N197 glycan into JR-FL trimer also leads to a ~4 fold reduction in b12 IC50 (Fig 6C), consistent with its role in a putative “glycan fence” that regulates access to the CD4bs [64,67]. In contrast to the 613 serum, the 647 serum was affected by mutations of residues D230, P238 and E269 in loops A and C of the C2 region that straddle a disulfide-bonded hairpin (Fig 6B and 6C). In this case, neutralization was not exclusively eliminated by the introduction of a new glycan at residue 230, but instead depended on the presence of an aspartic acid at this position. The resistance of all these mutants to the CO11 V3 mAb suggests that they retain a tier 2 phenotype. Therefore, we can conclude that these mutants directly affected serum recognition rather than having an adverse effect on trimer folding.

Taken together, the above mapping data suggest that both of our potent sera take advantage of the relatively sparse glycan coverage of the JR-FL C2 region. Notably, the N197 glycan is conserved in 98.6% viruses of a multi-clade panel of >4,000 sequences (Fig 6B). Further experiments using a set of JR-CSF Env-based chimeras (S11B Fig) revealed that two mutants (8073 and 8087) that eliminate the N197 glycan knocked in 613 serum neutralization sensitivity, suggesting that the N197 glycan regulates tier 2 cross-reactivity. These mutants exhibited increased b12 sensitivity [67], consistent with a role of N197 glycan in the glycan fence. In contrast, all JR-CSF-based chimeras remained insensitive to the 647 serum, suggesting that, in this case, neutralization is highly strain-specific and context dependent. An expanded set of JR-CSF-based chimeras similar to the JR-FL chimeras in S11B Fig were also tested, but none were sensitive to the 613 or 647 sera.

### Serum 613 exhibits N197-regulated tier 2 breadth within clade B

**Fig 7:**
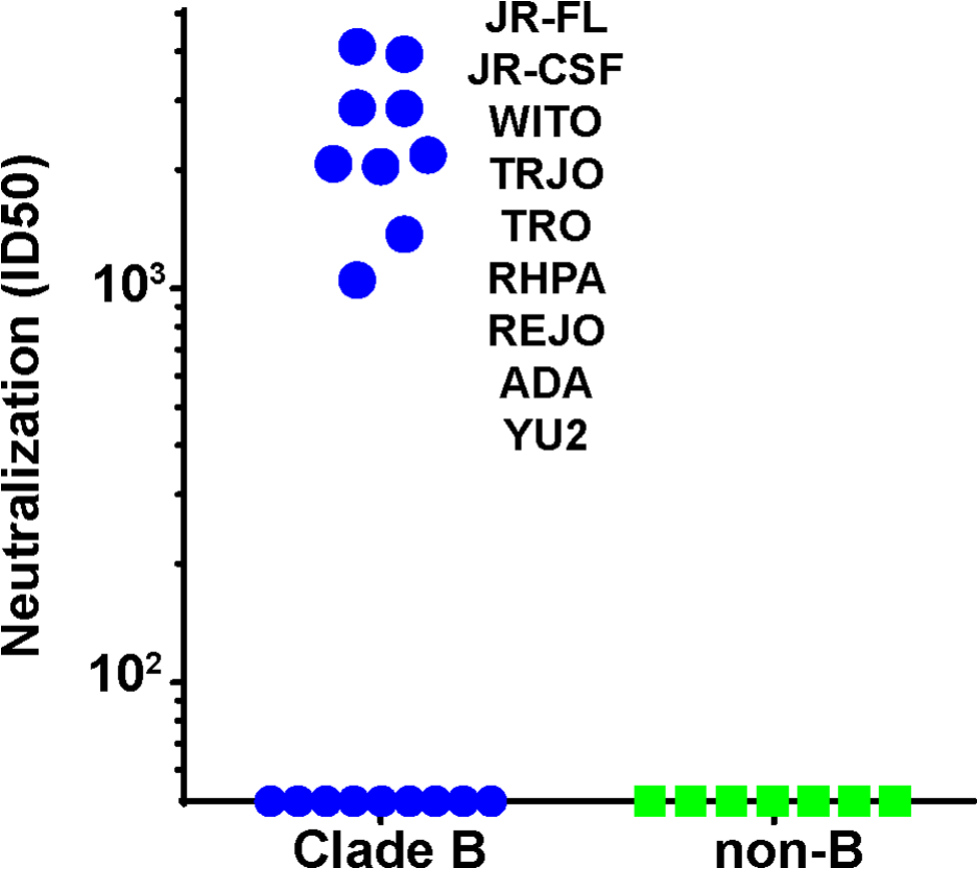
Serum 613 neutralization breadth of N197 knockout mutants. 613 serum ID50s against N197 glycan knockout mutants of various clade B (blue symbols) and non-clade B (green symbols) in the TZM-bl assay. N197 glycan knockout mutants sensitive to 613 serum are indicated. Raw data are shown in S5 Table.

We next asked if the N197 glycan regulated the sensitivity of other isolates to our sera, as it did for the JR-CSF isolate. To answer this question, we generated N197 glycan-deficient mutants in 28 other tier 2 isolates sampled from several clades, all of which resisted 613 serum neutralization in the presence of this glycan (S5 Table). Remarkably, the removal of the N197 glycan rendered 9 of these viruses (i.e., 31%) sensitive to the 613 serum (S5A Table and Fig 7). All 9 viruses were from clade B and constituted 50% of the clade B N197 knockout mutants tested (n = 18; Fig 7). Our other neutralizing trimer VLP vaccine serum (from animal 347) neutralized 3 of these 9 viruses (JR-FL, JR-CSF and ADA; S5A Table). Neither serum neutralized any of the 11 non-clade B viruses, which included Envs from clades A, C and several Chinese B' isolates [68]. Not surprisingly, the 647 serum did not neutralize any of the N197 mutants.

To properly interpret these findings, it was important to monitor any overt changes associated with the removal of the N197 glycan that might signify a loss of tier 2 phenotype and therefore may impact sensitivity to our sera. Accordingly, our mutants were further characterized using a panel of mAbs (S5 Table). These included 6 non-nAbs, 14e, 39F, b6, F105, 17b and 48d, directed to V3, CD4bs and CD4i epitopes. In addition, we monitored sensitivity to a weakly neutralizing HIV+ serum, BB68 [54]. We arbitrarily ascribed global sensitivity to any N197 mutant that was sensitive to at least 2 of the 6 non-nAbs. Six N197Q mutants (JR-CSF, ADA, SC422, YU2, PVO.4 and BaL) were thus found to be globally sensitive and were therefore excluded from further analysis (S5C Table). Nevertheless, since 5 of these mutants were sensitive to the 613 serum and 4 were sensitive to the 347 serum, we decided to remake 4 of them (JR-CSF, ADA, YU2 and PAVO) as N197D mutants in the hope that the different amino acid exchange would preserve a tier 2 phenotype. A N197D BaL mutant was not made, however, because the parent virus was sensitive to two of our non-nAbs (S5C Table). All 4 new N197D mutants retained a tier 2 phenotype. Moreover, JR-CSF and ADA mutants were sensitive to both the 613 and 347 sera, the YU2 N197D mutant was sensitive to the 613 serum and the PVO.4 mutant resisted both sera. Thus, all 9 613-sensitive mutants shown in Fig 7A were verified to have a tier 2 phenotype, consistent with modest tier 2 breadth within clade B by recognition of the surface protected by the N197 glycan. The 347 serum also exhibited modest breadth that might have been limited by the fact that it was taken at bleed 3 rather than bleed 4. Although this “breadth” depends on the absence of the N197 glycan, since all the mutant viruses retain a tier 2 phenotype, our data suggest that addressing the key problem of breadth in vaccine design may in future be possible.

### Neutralization fingerprint profiling of the 613 serum epitope

**Fig 8:**
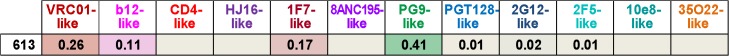
Fingerprint analysis of the 613 serum suggests weak associations with CD4 binding site and V2 quaternary epitopes. A fingerprint analysis [69] was performed based on the sequences of neutralized and non-neutralized N197 mutants of a subset of 24 mutants and 12 bnAb specificities (see methods). The selected N197 mutants (the parent Env Genbank accession number in parentheses) were 6101.10 (AAT36747), 7165 (AAW64252), 92US715 (AAB04079), AC10.29 (AAW64261), ADA.DG (AAR05843), BG1168 (AAW64258), BL01.DG (AAN39728), CNE12 (ADI62512), CNE4 (ADI62567), JR-CSF (AAB03749), JR-FL (AAB05604), PVO.04 (AAW64259), Q769.d22 (AAM66234), Q769.h5 (AAM66238), REJO4541.67 (AAW64264), RHPA.7 (AAW64262), TRJO (AAW64265), TRO.11 (AAW64260), WITO.33 (AAW64266), YU2.DG (M93258), ZA012.29 (ACF75939), ZM106.9 (AAR09562), ZM55.28a (AAR09381) and QH0515 (AAW64255). Delineation values >0.2 are considered to indicate the presence of particular specificities in the polyclonal serum.

We next measured the sensitivity of N197 mutant panel to various neutralizing ligands to determine any patterns that might partition with serum 613 sensitivity (S5 Table), using a previously published fingerprinting analysis [69]. Computational analysis suggests that strain selection can improve the predictive accuracy of this approach, i.e., the ability to discriminate between the different specificities. A recently developed method for virus panel selection was applied to determine any subsets (including all 9 613 serum-sensitive viruses) of the 29 member N197 mutant panel in S5 Table that are more suitable for the fingerprinting analysis compared to the full panel [69]. All possible panels of sizes 20–28 were evaluated. The best panel included 23 N197 mutant strains. Although this was preferable to the full 29-strain panel, neither were optimal and could therefore adversely affect the reliability of any predictions. Our panel size was limited by the practical challenge of making N197 mutants for each Env clone and performing a full neutralization analysis. In the fingerprinting analysis, neutralization via a particular epitope is typically associated with delineation values of >0.3, and preferably >0.4. However, we have previously confirmed positive signals of >0.2 [5,49,69]. In this case, a 23-strain panel and 12 bnAb specificities were used for fingerprint delineation. The 1F7 mAb was included because it is known to be N197-sensitive [67]. The strongest fingerprint signal for the 613 serum was for PG9-like antibodies (Fig 8). It may be no coincidence that all 9 of the 613-sensitive N197 mutants were also sensitive to at least one of the mAbs PG16, PGT145 and VRC26, whose epitopes depend on a compact quaternary conformation (S5 Table). This was not true for 4 of the 9 clade B 613 serum-resistant mutants (1168, QH0515.01, 6101 and BL01). Several non-B clade mutants also resisted these bnAbs. It should be noted that the 613 serum can neutralize the parent JR-FL strain in the absence of a lysine at residue 168 of the V2 loop that is critical for known V2 quaternary-dependent neutralizing mAbs. Therefore, in keeping with the marginal competition in Fig 5, it is unlikely that the 613 serum targets this epitope cluster. Rather, we suggest that trimer compactness is important for neutralization by both the 613 serum and by broadly neutralizing V2 quaternary mAbs.

Cumulatively, CD4bs nAbs also showed a strong fingerprint signal; VRC01-like signals were the strongest, along with detectable b12-like and 1F7-like signals (Fig 8). This is consistent with the solid 613 serum-mediated competition of CD4bs mAbs (Fig 5). Overall, N197 mutants exhibited a trend for slightly greater sensitivity to CD4 ligands. Specifically, 84.9% of virus-ligand combinations in which neutralization was detectable (146 of 172 virus-ligand combinations) exhibited a >2 fold increase in sensitivity. This is consistent with its role in the glycan fence that regulates CD4bs access. There were no consistent effects on several other bnAb epitopes analyzed in S5 Table.

Despite these observed PG9-like and CD4bs-like patternsof serum 613, none of the delineation signals were exceptionally strong. This is consistent with a unique epitope that does not fall entirely into any known category. Rather, it suggests a CD4bs-like quaternary epitope that exhibits some characteristics of both clusters. An alignment of the 18 clade B sequences derived from Fig 7 and S5 Table did not reveal any consistent elements (e.g. glycans, V1V2 length, CD4 contact regions) that partitioned with 613 serum sensitivity (S12 Fig). Similarly, an investigation of a variety of phylogenetic tree models (neighbor join, UPGMA, maximum parsimony) and substitution models (Poisson, p-distance) rooted to various reference sequences failed to identify any distinct clustering of 613-sensitive viruses, in part due to the low confidence of bootstrap values. Ultimately, high sequence variability defied any attempt to decipher common element(s) that might predict sensitivity. Perhaps the only clear conclusion we can make is that it is probably no coincidence that all the sensitive viruses were derived from clade B.

## Discussion

**Fig 9:**
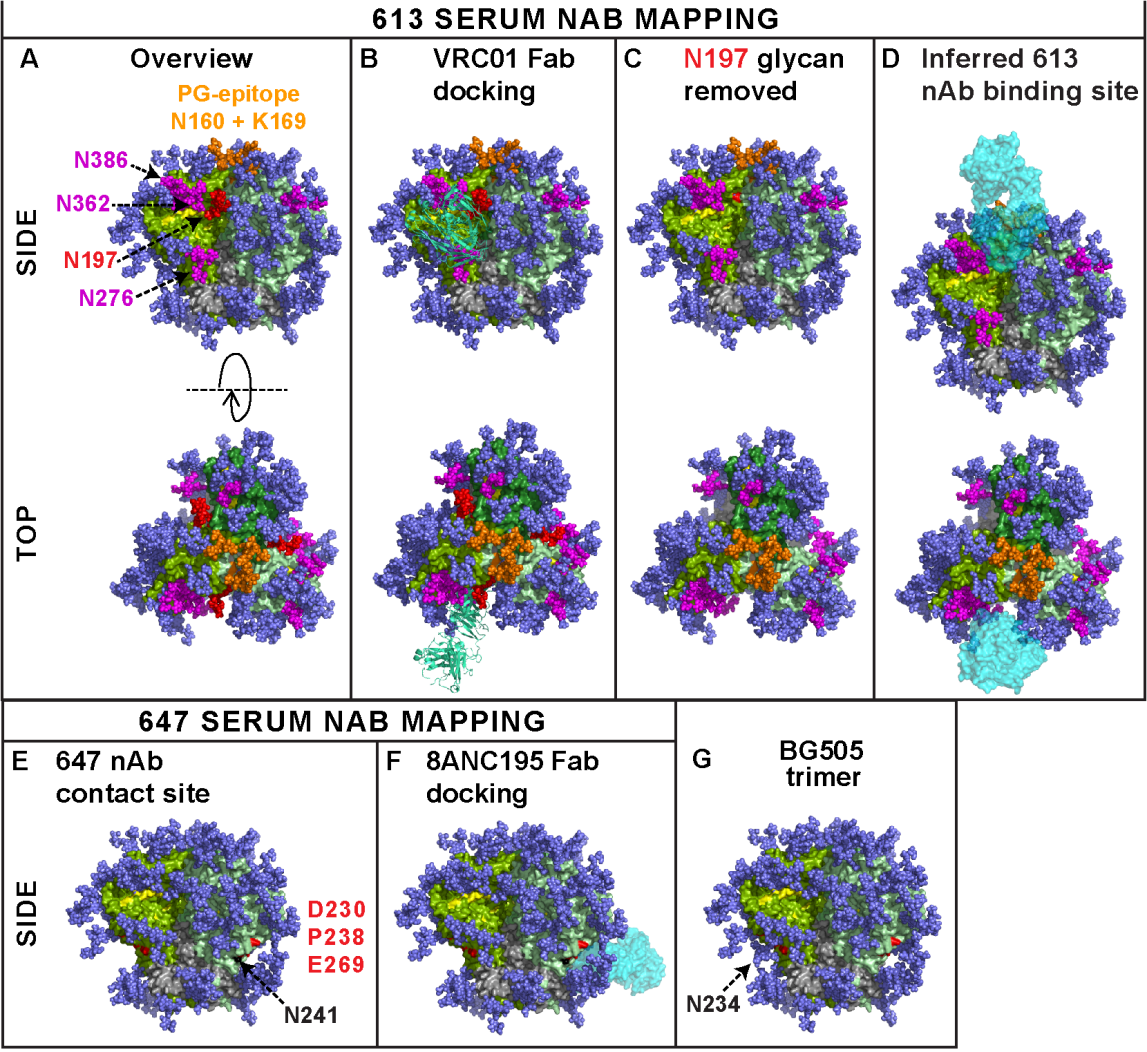
Modeling the epitope footprints of neutralizing sera 613 and 647. The binding sites of the two potently neutralizing rabbit sera 613 and 647 are modeled on BG505 SOSIP.664 Env trimers. Images were created from pdb 4TVP and show side and top views, as indicated. Gp41 is shown in metallic grey. Different gp120 protomers are shown in different shades of green so they can be discerned from one another. In nature, a significant proportion of glycans are complex in nature and may exhibit branches that are difficult to predict and/or unequivocally model [89]. Therefore, all glycans (mostly shown in dark blue) were rendered as Man_8_GlcNAc_2_. Since complex glycans usually have a greater mass than Man_8_GlcNAc_2_ (see Fig 1 of [64]), the glycan coverage shown here may be a slight underestimate compared to infectious spikes. On the other hand, this model employs all available sequons on the trimer, whereas in nature, close proximity may result in some sequons being unused [64]. A) The CD4 binding loop (residues 360–370) and other putative CD4 contacts at positions 427 and 477 are shaded yellow. A putative "glycan fence", consisting of glycans N197, N276, N362 and N386 is shown in red (for N197) and magenta. The N160 glycan and K169 residue contacts of PG cluster (V1V2 loop-specific) mAbs at the apex of the trimer are shown in orange. B) Fab VRC01 (3NGB), rendered in pea green ribbon format, is shown docked to the trimer. C) The N197 glycan is removed and the underlying N197 residue is labeled red. D) The putative binding site for the 613 serum rendered as a cyan Fab is modeled. E) Residues 230, 238 (loop A) and 269 (loop C) of the gp120 C2 region targeted by serum 647 are shown in red. The N234 glycan is omitted, as in the JR-FL strain. However, residue N241 is colored in black to indicate an additional glycan in the JR-FL strain that is absent in BG505. F) Fab 8ANC195 is docked on the structure in part E, as the best approximation of the 647 serum. G) The N234 glycan present in BG505 is added back, showing how it could clash with 647 nAbs.

Here, for the first time, we showed that authentic trimers can elicit potent nAbs directed to quaternary epitopes that take advantage of strain-specific holes in the glycan shield. This establishes a mechanism by which autologous antibodies can potently neutralize tier 2 isolates in a vaccine setting. The binding sites of two potent sera (from animals 613 and 647) are perhaps best visualized as footprints on the near native BG505.SOSIP.664 Env trimer model (Fig 9) [5,30]. Given the collective features of the 613 serum nAb—i) CD4bs overlap, ii) N197 glycan-sensitivity, iii) modest PGT cluster II overlap, iv) quaternary dependency, and v) CD4bs/PG9-like fingerprint—we suggest that it targets a CD4bs-like hole in the glycan shield that is accessible only on the trimer, possibly close to the gp120 protomer interface. To reach the CD4bs cavity, antibodies must navigate through a putative "glycan fence" [64], consisting of glycans N197 (in red in Fig 9) [70], N276 [10,32], N362, N386 (all in magenta), and possibly others, depending on the Env strain (Fig 9A). The difficulty of this challenge is illustrated by the exquisite docking of CD4bs mAb VRC01 on the trimer (Fig 9B). The 613 serum nAb must also navigate this fence, albeit with the N197 glycan missing in the JR-FL strain (modeled in Fig 9C and 9D).

Serum 647 nAbs make contact with loops A and C of the C2 region that straddle the inner and outer domains (labeled red in Fig 9E). In this BG505 trimer model, we labeled residue 241 in black to signify the presence of a glycan in the JR-FL trimer that happens to be absent in BG505 Env. Conversely, we removed a glycan at residue 234, consistent with its absence in JR-FL Env trimers. Like the 613 serum nAb, the 647 serum appears to take advantage of a rare glycan-deficient patch (modeled in Fig 9F). The position of the 647 epitope on the lower edge of the gp120 outer domain is compatible with a lateral angle of approach similar to that of recently reported mAb 8ANC195, which also targets the C2 hairpin and competes with CD4bs antibodies [47,71]. However, unlike the 647 serum, mAb 8ANC195 recognizes the common N234 glycan that is absent in JR-FL [72]. Even when this glycan was introduced into JR-FL Env, 8ANC195 neutralization remained undetectable and thus prevented us from being able to compete serum 647 and mAb 8ANC195 in the trimer VLP ELISA (as in Fig 5). This difference in specificity might in part explain 8ANC195's breadth, as compared to the strain-specificity of the 647 serum. The N234 glycan present in BG505 and 79.7% of 4,265 published strains is added back in Fig 9G, revealing a possible clash with 647 serum nAb binding. Overall, the epitopes of our 3 potent sera add to previous evidence that the JR-FL Env can quite frequently induce nAbs that overlap the CD4bs [12,13]. This may be a result of strain-specific gaps in its glycan armor of the gp120 C2 region that protects the CD4bs. Similar glycan-deprived sites can also be targets for autologous neutralization in natural infection [9,11].

The potent nAb responses in rabbits 613 and 347 far exceeded any we observed in previous VLP immunogenicity studies [28,50]. This may be in part due to improvements that were suggested by these earlier studies: i) that high doses may be important, ii) that rabbit models may be more competent as nAb vaccine models, and iii) that it may be important to refocus responses on the native trimer by eliminating antigenic interference by non-functional Env. The use of more efficient expression methods should in future help us to ensure that doses are sufficient to induce maximum responses. Our newly adopted Gag-only plasmids (as compared to subgenomic NL-Luc) are also safer because they lack a Ψ packaging signal and therefore do not carry a genetic payload [73]. In contrast to rabbits, the relatively high gp120 binding (Fig 2) and tier 1 nAb titers (S3 Table) but lack of tier 2 nAbs (Fig 2) in guinea pigs suggest that VLPs are immunogenic, but that this species struggles to target the native Env trimer and instead target degraded forms or Env that may appear later.

Different stochastic processes might underlie the inconsistent nAb development to the various immunogens we tested. In the case of the gp120 prime-protein boost regimen used in the 2922 animal, since monomeric gp120 is not an authentic target, it may only rarely induce antibodies that happen to cross-react with native trimers and instead largely induces ‘off target’ responses. In the case of regimens that involve authentic trimer as an antigen, different challenges come into play, principally that it is a complex antigen that presents few opportunities for antibody development. For this reason, tier 2 nAbs developed in only 1 of 20 animals that received a DNA prime that expresses largely authentic Env trimer (S1 Table). An additional factor may have been this plasmid DNA expresses only modest amounts of trimer to ensure a mature, authentic product, but that could adversely affect the robustness of priming. Paradoxically, however, the rare tier 2 nAbs observed in animal 647 were associated with unusually effective DNA priming, as evidenced by an exceptional ID50 to the MN strain (S1 Table). The distinct specificities of the tier 1 and 2 nAbs in this animal (S7 Fig) suggest the development of separate antibody lineages: potent tier 1 nAbs that recognize non-native forms of Env are produced during DNA priming, as well as trimer-specific tier 2 nAbs that are expanded upon the first protein boost. Clearly, this effect may be difficult to replicate with any reliability. In contrast, our data show that trimer VLPs induce only limited tier 1 nAbs and generated tier 2 nAbs more frequently, albeit still in a minority of vaccinees. This inconsistency may relate to limited ability of small animal antibody repertoires to find solutions for binding this complex antigen—a problem that may be exacerbated by the low Env spike density on VLP surfaces that may limit crucial signals necessary for affinity selection [31,74]. Overall, these observations suggest that, despite the exposed protein patch in the C2 region, the native JR-FL trimer remains poorly immunogenic.

Clearly, it will be important to try to improve the consistency of vaccine nAb responses. One approach may be to combine the approaches used here in DNA prime-VLP boost regimens [13,20,22,75]. A more intriguing approach might be to engineer additional holes in the glycan shield to encourage nAb development [32,76]. For example, CD4bs nAb development might be facilitated by selectively removing glycan fence posts to avoid clashes [32,67,70]. One concern with this strategy is that glycan-depleted trimers may be overtly sensitive to non-neutralizing antibodies, as occurs with the removal of the N301 glycan [64,77]. In addition, some glycans cannot be removed without adversely affecting trimer folding [29]. Nevertheless, in our experience, most single glycans can be removed without global effects on trimer folding and maintain a tier 2 phenotype (i.e., non-neutralizing epitopes remain occluded). In fact, the conservation and use of glycosylation sites vary considerably between isolates. It is fairly common for tier 2 isolates to lack one or two glycans, like JR-FL, that are present on the majority of other circulating tier 2 viruses. Indeed, modestly glycan-depleted tier 2 viruses can in some scenarios transmit infection, perhaps because these variants can facilitate acquisition in settings where nAb resistance is unimportant [8,78–80].

To our knowledge, our data provide the first clear evidence of potent, vaccine-elicited nAbs directed to a reasonably well-conserved site on tier 2 viruses—albeit one that is camouflaged by a glycan on most strains. In contrast, recent evidence suggests that broad HIV-1 neutralization almost always *requires* glycan recognition—as opposed to other viruses such as influenza, where, despite high glycan context, no glycan recognition occurs [5]. Our results therefore show how effective the glycan shield is in preventing the development of broad neutralization. With this in mind, how might we try to induce cross-reactive tier 2 nAbs in the future? One strategy might be to evolve breadth from initial autologous nAbs using modified boosts. After all, in both vaccine and natural infection settings, initial nAbs are invariably autologous and there may be no practical way to elicit bnAbs directly. The mechanisms of nAb breadth development in natural infection provide model scenarios that might be mimicked by vaccine strategies [8,9,11,81–85]. For example, breadth sometimes develops as a result of virus escape from autologous nAbs in which a newly introduced glycan creates a new broad site of vulnerability [8–11]. Indeed, while glycans are largely immunosilent, bnAbs can eventually develop that recognize composite epitopes involving glycan contacts. The evolutionary changes in nAb specificity needed to accommodate a newly added glycan might be either to avoid clashes, perhaps by altering the angle of approach to its epitope (e.g. CD4bs [86]) or to incorporate the glycan as part of its epitope. Either of these scenarios might be a useful first step towards cross-reactivity. For example, it might be possible to evolve changes in neutralizing antibodies like those observed in animal 613 by using appropriate boosts to encourage the development of antibody variants that are no longer sensitive to the N197 glycan.

In summary, this study introduces some encouraging new concepts for HIV-1 nAb vaccine development. Specifically that i) native trimers presented *in situ* can induce tier 2 nAbs, ii) nAbs recognize quaternary epitopes, iii) nAbs target vulnerable gaps in the glycan shield, and iv) these nAbs can neutralize other tier 2 viruses provided that appropriate gaps are artificially introduced in their glycan shell. The advances we made here with VLP immunogens required us to address various limiting factors we identified in preceding studies. A continued commitment to further iterative, rational improvements may in future bring us closer to our goal of inducing tier 2 breadth.

## Materials and Methods

### Anti-HIV-1 Env monoclonal antibodies

Monoclonal antibodies (mAbs) were obtained from their producers, the AIDS reagent repositories of the UK Medical Research Council and the NIH, or were purchased from commercial suppliers. Further information on these mAbs can be found at the web link: (www.hiv.lanl.gov). The mAb panel included the following (originators given in parentheses): 2G12 (Katinger), directed to a unique glycan-dependent epitope of gp120 [87]; 14e, 39F and CO11 (J. Robinson), directed to the gp120 V3 loop [25,28,53]; b12 and b6 (Burton), VRC01 and VRC03 (Mascola), 8ANC131 and 3BNC117 (Nussenzweig), CH103 (Haynes), HJ16 (Lanzavecchia), F105 (Posner) 15e (J.Robinson) and 1F7 (Katinger), directed to epitopes that overlap the CD4bs [25,38,40,67,72,83]; 8ANC195 (Nussenzweig), directed to the C2 region [47,71]; 17b and 48d (J.Robinson), directed to CD4-induced (CD4i) epitopes of gp120; PGT121, PGT125 and PGT128 (Burton) directed to epitopes involving the base of the V3 loop of gp120 and the N332 glycan [42]; PG9, PG16 and PGT145 (Burton), and CAP256-VRC26.08 (abbreviated as VRC26; directed to quaternary, glycan-dependent epitopes that involve the V2 loop [37,56,84]; 35O22 (Connors), directed to a quaternary epitope of the gp120-gp41 interface [49]; PGT151 (D. Burton), directed to a quaternary gp41 epitope [43]; 7B2 and 2.2B (J. Robinson), directed to the gp41 cluster I and II epitopes, respectively [11]; 4E10, 2F5 (Katinger) and 10e8 (Connors), directed to the gp41 membrane-proximal ectodomain region (MPER) [45].

### Recombinant gp120 monomer, gp41, soluble CD4 and CD4-IgG

Recombinant monomeric JR-FL gp120 produced in CHO cells and soluble CD4 (sCD4) consisting of its 4 outer domains were gifts from Progenics Pharmaceuticals (Tarrytown, NY). 2-domain CD4-Ig was a gift from Marie Pancera (NIH). Recombinant HXB2 gp41 was obtained from Meridian Life Science (Catalog#VTI310; residues 546–682, Saco, Maine).

### Plasmids and mutagenesis

Plasmid pCAGGS was used to express JR-FL gp160∆CT on VLP surfaces [51,55]. Gp160∆CT is truncated at amino acid 709, leaving a 3 amino acid gp41 cytoplasmic tail. This increases native trimer expression and can be used to produce pseudoviruses with similar neutralization sensitivity profiles compared to their full-length gp160 counterparts [53]. The use of the JR-FL Env strain has several rare advantages, including efficient expression and gp120/gp41 processing [51,53]. Mutants were generated by Quikchange (Agilent Technologies) and were numbered according to the HXB2 reference strain [54]. "SOS" mutations (A501C and T506C) introduce an intermolecular disulfide bond between gp120 and gp41 [57]. E168K and N189A mutations knock in the broadly neutralizing “PG” epitopes that are normally absent in the JR-FL isolate and increases trimer expression [25]. The A328G mutation dramatically enhances neutralization sensitivity, otherwise known as "global" sensitivity [25]. The D368R mutation abrogates CD4 binding capability [13].

Various plasmids expressing other Env gp160s were obtained from the NIH AIDS repository, including clade B and C virus panels. We also obtained a set of Chinese B' Env-expressing plasmids from Dr. Zhiwei Chen [68]. Mutant versions of these sequences in which the N197 glycan is eliminated were used for the fingerprinting analysis. Codon optimized soluble JR-FL D368R gp140 foldon (gp140F) was expressed from a pCDNA3.1(-) vector using a CD5 leader and CMV promoter [13]. Another set of plasmids encoded a series of domain-exchanged hybrid gp160s using sequences from the JR-FL and JR-CSF isolates [20,67].

Other plasmids used to make VLPs included Env-deficient sub-genomic plasmids pNL4-3.Luc.R-E- and pSG3∆Env that have been described previously [50]. Plasmids pMV-2024 and pMV-0932 express full-length SIVmac251 (BK28) Gag and codon-optimized HIV-1 Rev, respectively, both under the control of a CMV promoter.

### VLP production

First generation VLPs were produced by co-transfecting 293T cells with an Env-expressing plasmid and pNL4-3.Luc.R-E-, using polyethyleneimine, as described previously [25]. Second generation VLPs were produced using plasmids pMV-2024 and pMV-0932 in place of pNL4-3.Luc.R-E-. Two days later, supernatants were collected, precleared by low speed centrifugation and pelleted at 50,000 x g in a Sorvall SS34 rotor. To remove residual medium, VLP pellets were diluted with 1ml of PBS, then re-centrifuged at 15,000 rpm and resuspended in PBS at 1,000 x the original concentration. VLPs were referred to as WT-VLPs or SOS-VLPs, depending on the form of Env displayed on their surfaces or as bald-VLPs, bearing no Env, produced by transfecting pNL4-3.Luc.R-E- alone [50]. "Trimer-VLPs" were made by digestion using a cocktail of proteases including proteinase K, subtilisin, trypsin and chymotrypsin, as previously described [25,52]. VLPs were inactivated using aldrithiol (AT-2) [50].

### Animal immunizations

#### Species

Dunkin Hartley guinea pigs and New Zealand white rabbits in groups 1–4 were housed and immunized at the Pocono Rabbit Farm (Canadensis, PA)—a site that has been approved by the Association for Assessment and Accreditation of Laboratory Animal Care (AALAC). All animals were fed, housed and handled in strict accordance with the recommendations of the NIH *Guide for the Care and Use of Laboratory Animals* and the Animal Welfare Act.

All immunization protocols for rabbits and guinea pigs were approved (protocol PRF2A) by the Explora Biolabs Animal Care and Use Committee (IACUC). Explora Biolabs’ animal welfare assurance (AWA) number is A4487-01. Pocono Rabbit Farm, Covance and Aldevron are all approved for rabbit and guinea pig immunizations by the Association for Assessment and Accreditation of Laboratory Animal Care (AALAC). The AWA number Pocono Rabbit Farm is A3886-01. For all immunization and bleed protocols, pain and distress was slight and momentary and did not affect animal health. Discomfort and injury to animals was limited to that which is unavoidable in the conduct of scientifically valuable research. Analgesics, anesthetics, and tranquilizing drugs were used as necessary by veterinary staff. After the completion of all immunizations and bleeds, rabbits were euthanized by injection of an overdose of anesthesia according to NIH guidelines. Ketamine and xyalzine were administered intramuscularly at 35mg/kg and 5mg/kg, respectively, followed by exsanguination via cardiac puncture.

Procedures for adverse reactions to immunization followed established practices that include measures for dealing with unexpected outcomes. For example, if an animal had an adverse reaction at the injection site, depending on the severity, the response would be either i) none in the case of mild reactions, ii) anesthetic in the case of medium reaction, and iii) veterinary staff would be brought in for severe cases, in which it may be necessary to euthanize via an overdose of pentobarbital (100mg/kg i.v.), according to guidelines established in 1993 by the American Veterinary Medical Association Panel on Euthanasia. However, in this study, reactions to all procedures were only mild and no animals were euthanized over the course of immunizations and bleeds. One rabbit, however (347) died unexpectedly, but this was considered to be unrelated to the immunization and bleed procedures.

#### Immunization

All rabbits and guinea pigs were female. Three groups of 4 rabbits and one group of 4 guinea pigs were immunized intramuscularly with VLPs, as indicated in groups 1–4 of Fig 1. VLPs were formulated in AS01_B_ (Glaxo SmithKline, consisting of liposomes containing deacylated monophosphoryl lipid A and QS-21). VLP doses were determined by documenting their relative antigenicity compared with JR-FL gp120 by ELISA, using various mAbs, as we described previously [28]. Immunizations occurred at weeks 0, 4, 12 and 20. Standard serum volumes were drawn on the day of each immunization and two weeks thereafter.

#### Group 5 immunization studies

Group 5 rabbit sera (Fig 1) were sourced from different vaccination projects that used various JR-FL Env-based DNA prime-soluble protein boost protocols. Animal 647 was one of 4 animals immunized intramuscularly 3 times at weeks 0, 4 and 8 by electroporation using an Ichor device with 500μg non-codon-optimized pSVIII JR-FL SOS gp160∆CT plasmid DNA and 25μg of pCTat. The moderate expression by these plasmids was previously shown to result in largely native Env trimer expression on transfected cell surfaces [13]. Four other groups of 4 rabbits were primed 3 times with pSVIII JR-FL WT gp160∆CT, pSVIII JR-FL SOS gp160∆CT E168K, pSVIII JR-FL SOS gp160∆CT T569A+I675V, or no DNA (S1 Table). The I569T+A675V mutant imparts a “globally sensitive” (i.e. tier 1) phenotype [62]. All 20 rabbits (5 groups of 4) were later boosted at weeks 20 and 24 with 100μg JR-FL gp140 foldon (gp140F) trimers that had been expressed and purified as described previously and formulated in Adjuplex (Advanced BioAdjuvants, consisting of purified lecithin and carbomer homopolymer) [13]. Bleeds were taken 2 weeks after each immunization. This work was performed at Covance (Denver, PA), an AALAC-accredited site under protocol 0310–11 that was approved by the Covance Research Products IACUC Committee (AWA number A3850-01).

Serum from rabbit 2922 of group 5 was selected from a group of 10 female New Zealand white injected intramuscularly using an Advisys electroporation device with 400mg of a pMAmp plasmid DNA construct encoding codon-optimized JR-FL gp120, and then boosted with a JR-FL gp120 monomer protein purified from CHO cells adjuvanted with AS02_A_ (Glaxo SmithKline, consisting of monophosphoryl lipid A and QS-21 in an oil and water emulsion) [20]. This work was carried out at Aldevron LLC (Fargo, ND) under protocols 2-04-007-09-2005, 1-06-001-08-2007, and 1-07-005-10-2008 that were approved by the Aldevron IACUC Committee; AWA number A4422-01). Rabbits were immunized with DNA on days 0, 28 and 56, boosted with protein on day 84, and terminal bleed used in this study were collected on day 98.

The 7672 serum of group 5 was described in a previous report [59], derived from a rabbit immunized with "G2C" mutant gp120 in DNA prime-boost format, using Ribi Ras3C as an adjuvant in boosts. The G2C mutant involves a graft of the MPER region into the gp120 V2 loop. The 849 serum of group 5 was also described in a previous report [12,58] on rabbits immunized with a JR-FL SOSIP gp140 DNA prime, followed by a JR-FL SOSIP gp140 trimer boost in QS-21/Ribi Ras3C. This was a "first generation" SOSIP immunogen, featuring a I559P mutation in gp41 subunit to stabilize soluble SOS gp140 trimers.

### Reference sera and plasmas

Reference controls included a pooled serum generated from six rabbits that were immunized with monomeric gp120 in AS01_B_, as described previously [28]. HIV-1-infected donor plasmas BB34, BB68, 1686, 1702 and N160 and uninfected control plasma 210 were also described previously [28,54].

### ELISAs using recombinant gp120, gp41 and VLPs

ELISAs were used to measure serum binding to various antigens and were also used to determine their specificities [25,28,60]. Briefly, Immulon II plates were coated with 20x concentrated VLPs, recombinant gp120 or gp41 at 5μg/ml overnight at 4°C. Following a PBS wash and blocking, sera were titrated against each antigen in blocking buffer. Species-specific alkaline phosphatase anti-Fc conjugates (Accurate, Westbury, NY) and SigmaFAST p-nitrophenyl phosphate tablets (Sigma) were then used to detect binding. Plates were read at 405nm. Titers are taken when ELISA signals exhibited an optical density of 0.5 (approximately 3x background).

In competitive ELISAs, we determined the ability of sera at a 1:10 dilution to inhibit binding of graded doses of various biotinylated mAbs to SOS E168K trimer VLPs. MAbs were biotinylated using NHS-X-biotin reagent (Calbiochem). Biotinylated mAb binding was detected using streptavidin-alkaline phosphatase (Vector, Burlingame, CA), and developed as above. A prebleed rabbit serum or an HIV-1-seronegative plasma (donor 210) were used as reference controls. Titers of biotinylated mAbs (in μg/ml) measured at OD = 0.5 (approximately 3 x background) were determined in the presence of competitor and control samples. Then, competition was expressed as the % residual binding = [(titer in the presence of the control sample)/(titer in the presence of competitor) x 100].

### Blue native PAGE (BN-PAGE)-western blots

Blue native PAGE (BN-PAGE) was performed as described previously [3,50,51,53]. Briefly, VLPs were solubilized in 0.12% Triton X-100 in 1 mM EDTA. An equal volume of 2x sample buffer (100 mM morpholinepropanesulfonic acid (MOPS), 100 mM Tris HCl, pH 7.7, 40% glycerol, and 0.1% Coomassie blue) was added. Samples were then loaded onto a 4–12% Bis-Tris NuPAGE gel (Invitrogen) and separated at 4°C for 3 hours at 100V. The gel was then blotted onto polyvinylidene difluoride membrane, destained, immersed in blocking buffer (4% nonfat milk in PBS) and probed with an anti-gp120 cocktail (mAb b12 and 39F at 1μg/ml) and/or a anti-gp41 cocktail (mAb 2F5, 4E10, 7B2, 2.2B at 1μg/ml). Blots were then probed by an anti-human Fc alkaline phosphatase conjugate (Accurate Chemicals) and developed using SigmaFast BCIP/NBT substrate (Sigma).

BN-PAGE “shift” assays were used to measure the ability of antibodies to bind and deplete the unliganded trimer [3,25,50,51,53]. VLPs were incubated with mAb or serum for 1h at 37°C, then washed with PBS and resolved by BN-PAGE-Western blot, as above.

### VLP decay experiments

Experiments were conducted to determine the stability of VLPs over time after digestion with proteases.

#### Infectivity decay

NL-Luc/WTE168K+N189A WT VLPs were concentrated to 1000x from the original transfection supernatants. Half of the preparation was protease digested and the other half was mock incubated. Following a wash to remove proteases, VLPs were resuspended at a 20x final concentration then incubated at 4°C or 37°C. Samples were taken at intervals over the course of the total 72h incubation and measured for infectivity of CF2Th.CD4.CCR5 cells via a luciferase readout, according to the protocol described below for CF2 cell neutralization assays, but omitting antibodies. t_1/2_ values were calculated by interpolating the infectivity values at 0 hours and 72 hours.

#### Decay of trimer visualized by BN-PAGE

The stability of Env trimer on protease digested or undigested 2nd generation SIV p55/SOS E168K/Rev VLPs was monitored by BN-PAGE/Western blot. VLPs recovered at 1,000x were or were not digested and then were washed and resuspended at a 20x final concentration in PBS and then incubated at 4°C or 37°C. Samples were taken at intervals over the course of the total 72 hours of incubation for analysis by BN-PAGE. Upon harvesting, VLPs were pelleted and resuspended at 1,000x. The resulting pellet and 3 fold dilutions were analyzed by BN-PAGE-Western blot.

### Neutralization assays

Heat-inactivated sera and protein A-purified serum IgGs were analyzed for neutralization of various pseudoviruses produced by co-transfecting either 293T cells [64] with an Env plasmid and pNL4-3.Luc.R-E- (CF2 assays) or pSG3∆Env (TZM-bl assays). Data is representative of at least 3 repeat assays performed in duplicate.

#### Neutralization assays using CF2 cell targets

Neutralization assays using canine CF2 cells expressing CD4 and CCR5 receptors (CF2Th.CD4.CCR5) have been described previously [53]. Briefly, virus was incubated with graded dilutions of mAb or serum for 1 h at 37°C. The mixture was then added to CF2 cells, spinoculated at 300 x g for 15 min, incubated at 37°C for either 2 h (“washout” protocol), after which the medium was changed or for 3 days without a change of medium (“leave in” protocol). For SOS viruses, after 2 h incubation with target cells, infection was activated by adding 5mM DTT for 5 minutes, followed by a wash [53]. The cells were cultured a further 3 days, then luciferase activity was measured.

#### Post-CD4/CCR5 assay using CF2 cells

To measure neutralization activity after receptor engagement, SOS-VLPs were allowed to attach to CF2Th.CD4.CCR5 cells for 2 h at 37°C. Unbound VLPs were washed away, and titrated mAbs or serum were added, followed by 1 h incubation. As mentioned above, infection was then initiated by a 5 minute exposure to 5mM DTT [53].

#### Neutralization assays using TZM-bl cell targets

Neutralization assays using HeLa-derived TZM-bl cells (CD4 and CCR5 positive) were used as described previously [54]. Briefly, trypsinized TZM-bl cells were added to virus-serum mixtures and left for 3 days without a change of medium. Luciferase activity was measured at the end of 3 day incubation.

#### Neutralization assays by Monogram PhenoSense assay

PhenoSense neutralization assays were performed as described previously using proprietary materials and methods at Monogram [20]. Briefly, pseudoviruses were incubated with sera for 18h at 37°C before adding U87.CD4.CCR5.CXCR4 cells. Luciferase activity was measured at the end of 3-day incubation.

#### Soluble Env interference of neutralization

In modified neutralization assays, fixed concentrations (~1μg/ml) of recombinant JR-FL gp120 monomer or gp140 foldon (gp140F) trimer, each with D386R mutations to eliminate cellular CD4 binding, were added to the virus-antibody mixture to test for their ability to interfere with the neutralizing activity in the test serum or mAb [13].

#### Serum adsorption to Env-expressing cells

Protein A-agarose (Pierce-Thermo) purified serum IgG and mAbs were adsorbed against transfected 293T cells expressing SOS E168K+N189A Env, adapting a previously reported protocol [44]. In preparation for adsorptions, purified serum IgGs were adjusted to a volume of 500μl of PBS at 2mg/ml, corresponding to approximately a 1:5 serum dilution. An equal volume of each purified serum IgG was left unadsorbed for later neutralization analysis. MAbs VRC03 and 2G12 were adjusted to 20μg/ml. All samples were added to tubes of 5x10^7^ SOS E168K+N189A Env-expressing 293T cells in microtubes. Each was mixed thoroughly, incubated on ice for 20 minutes, then microcentrifuged at top speed. Supernatants were next collected and transferred to fresh tubes containing 5x10^7^ SOS E168K+N189A Env-expressing 293T cells for a second round of adsorption. This process was repeated for a total of 5 adsorptions. After adsorptions were complete, each supernatant was filtered and IgG was purified on protein A-agarose and then adjusted to the original input volume of 500μl. The resulting pre- and post-adsorption IgG samples were then compared for their neutralizing activities. To assess the extent to which IgG may have been lost or adsorbed during the adsorption process, IgG was assayed by reducing SDS-PAGE and then stained using a silver snap stain kit (Pierce-Thermo).

### Statistics

Mann-Whitney and ANOVA two-tailed tests were used to determine any statistically significant differences between study groups.

### Neutralization fingerprinting

The neutralization sensitivities of a panel of N197 glycan knockout mutants to a vaccine serum and a panel of neutralizing antibodies was used as a basis for a bioinformatics-based approach to inferring the neutralizing specificities in the serum, according to a previously published protocol [5,49,69,72]. A cutoff of ID50 = 50 (since input data was ‘<50’ in most cases) was used to denote the presence or absence of neutralization. A reference set of 12 antibody specificities was used in the analysis, with one or more representative antibodies included for each specificity: VRC01-like (VRC01, 3BNC117, 8ANC131, CH103), b12-like (b12), CD4 (sCD4, CD4-Ig), HJ16-like (HJ16), 1F7-like (1F7), 8ANC195-like (8ANC195), PG9-like (PG9, PGT145), PGT128-like (PGT121, PGT128), 2G12-like (2G12), 2F5-like (2F5), 10E8-like (4E10, 10E8), 35O22-like (35O22).

### Molecular modeling

The recently reported structure of BG505 SOSIP gp140 trimer ([5]; PDB id: 4TVP) was used to generate a visual model for the distribution of glycans on the native spike. First, atomic clashes present in the 4TVP crystal structure were relieved and missing side-chains rebuilt, by executing 1,000 symmetric ROSETTA-fixbb simulations, selecting the lowest scoring model, and then running a constrained ROSETTA-relax simulation. Each N-linked glycosylation motif for each of the 15 models was decorated with Man_8_GlcNAc_2_ glycans at sites of predicted oligomannose-type glycans and with Man_5_GlcNAc_2_ glycans at remaining sites. GlycanRelax [88] was used to approximate the conformational behavior of glycans in a glycoprotein context. For each model, 10 separate GlycanRelax trajectories of 10,000 cycles of MonteCarlo trials were carried out. Each glycan on the gp120 was allowed to move independently throughout the GlycanRelax minimization. A single low energy model was chosen for Figs. All Figs were generated in PyMOL Molecular Modeling Software (Version 1.5.0.4 Schrödinger, LLC).

### Ethics statement

The archived adult human plasmas used in this study have previously been described [28,54]. All donors provided written consent for the use of these samples. Institutional Review Board (IRB) approval for this project was obtained through the San Diego Biomedical Research Institute IRB Committee (approval number: IRB-14-04-JB; Federal Wide Assurance number: 00021327).

All immunization protocols for rabbits and guinea pigs were approved (protocol PRF2A) by the Explora Biolabs Animal Care and Use Committee (IACUC). Explora Biolabs’ animal welfare assurance (AWA) number is A4487-01. Pocono Rabbit Farm, Covance and Aldevron are all approved for rabbit and guinea pig immunizations by the Association for Assessment and Accreditation of Laboratory Animal Care (AALAC). The AWA number Pocono Rabbit Farm is A-3886-01. The protocol for rabbits immunized at Covance (Denver, PA), was approved (protocol 0310–11) by the Covance IACUC Committee (AWA number A-3850-01; NIH profile number 9640807). Protocols for rabbits immunized at Aldevron LLC (Fargo, ND) were approved (protocols 2-04-007-09-2005, 1-06-001-08-2007, and 1-07-005-10-2008) by the Aldevron IACUC Committee; AWA number A4422-01). All animals were fed, housed and handled in strict accordance with the recommendations of the NIH *Guide for the Care and Use of Laboratory Animals*, the Animal Welfare Act and Regulations and guidelines established in 1993 by the American Veterinary Medical Association Panel on Euthanasia. Two rabbit sera (7672 and 849) were archived from previous studies in which animal welfare information was documented [12,58,59].

## Supporting Information

S1 Fig Env expression on the surfaces of VLP immunogens. Env liberated from various VLPs was investigated by BN-PAGE-Western blot, probing with a cocktail of α-gp120 and α-gp41 mAbs. Lanes corresponding to the immunogens using in animal groups 1–4 are indicated. Ferritin was used as a molecular weight marker. Major bands are identified by cartoons representing (from top to bottom) native trimers, monomeric gp160, trimeric and monomeric gp41 stumps.
(TIF)

S2 Fig Stability of Env trimers on trimer VLPs. A) Infectivity decay of digested or undigested 1^st^ generation WT E168K+N189A VLPs was measured over time at 4°C and 37°C. B) BN-PAGE-Western blot analysis of the decay of undigested (upper panel) and digested (lower panel) 2^nd^ generation SOS E168K trimer VLPs at 37°C over time. Major bands are identified by cartoons that represent the native trimer and monomeric UNC gp160.
(TIF)

S3 Fig Statistical comparison of rabbit VLP serum binding titers between vaccine groups. A) Monomeric gp120, B) gp41 and C) bald VLP binding titers were compared between the current groups of rabbits (1, 2 and 4) and those of our previous study (Group R2-I in Fig 5 of ref. [28]). Mean titers are indicated by horizontal lines. Asterisks indicate significant differences (p<0.05) by Mann-Whitney two-tailed test.
(TIF)

S4 Fig Dose-dependent tier 2 neutralization by key rabbit vaccine sera. Representative sera from each animal group were titrated against A) JR-FL SOS E168K pseudovirus in the CF2 assay and B) JR-FL WT E168K pseudovirus in the TZM-bl assay.
(TIF)

S5 Fig Statistical comparison of VLP serum tier 1 nAbs between vaccine groups. Mean JR-FL A328G tier 1 nAb titers were compared in all 4 current groups of VLP sera and in groups of guinea pig (G2-I) and rabbit (R2-I) sera from our previous study [28]. Statistical significance is indicated with p value arising from an ANOVA one-way comparison between all groups.
(TIF)

S6 Fig Tier 1 and tier 2 nAb titers do not correlate in vaccine sera. Vaccine serum nAb titers against the tier 2 parent JR-FL E168K virus and tier 1 JR-FL A328G virus measured in CF2 cells were compared in a scatterplot. Filled symbols depict those with detectable tier 2 nAbs. Open symbols are from sera that lacked detectable tier 2 nAbs, which are arbitrarily assigned with titers of 1:4. An r^2^ and *p* value was calculated for all the data using linear regression best-fit line.
(TIF)

S7 Fig Soluble Env does not interfere with the tier 2 neutralizing activities of potent vaccine sera. Effect of adding 10μg/ml of purified D368R mutant versions of JR-FL monomeric gp120 (parts A and C) and gp140F trimer (part B) on serum and mAb neutralization of JR-FL gp160∆CT WT E168K (parts A and B) and JR-FL gp160∆CT WT A328G (part C) in the TZM-bl assay.
(TIF)

S8 Fig Standard errors of vaccine serum-mAb competitions in trimer VLP ELISA. This data partners with competition data in Fig 5.
(TIF)

S9 Fig Evaluation of mAb-mAb binding relationships by trimer VLP ELISA. A) As in Fig 5, competition data are shown as percentages; B) the standard errors of data in part A) are shown.
(TIF)

S10 Fig Dose dependent effects of mAbs, vaccine sera and human plasmas on biotinylated mAb binding to trimer VLPs. Here we show the effects of competitor antibodies on the binding of biotinylated mAbs over a range of concentrations.
(TIF)

S11 Fig Serum neutralization of JR-FL-JR-CSF chimeras. Chimeras comprising of a A) JR-FL Env or B) JR-CSF Env background with JR-FL/JR-CSF domain swaps, color coded as indicated, were evaluated for their infectivity (in relative light units; RLU) and sensitivity to vaccine sera and mAb CO11 and b12 neutralization.
(TIF)

S12 Fig Alignment of serum 613-sensitive and—resistant clade B Env N197 mutants. Amino acid sequences of clade B N197 mutant viruses from S5 Table were aligned by Tcoffee method using JalView software [90]. Only Envs from clade B were aligned and partitioned into two sections: the top being 613 serum-sensitive, the bottom being 613 serum-resistant. N197 glycan position is highlighted with red box. The parent clade B Env protein Genbank accession number are as follows: JR-FL (AAB05604), JR-CSF (AAB03749), WITO (AAW64266), TRJO4551 (AAW64265), TRO.11 (AAW64260), RHPA.4259 (AAW64262), REJO4541 (AAW64264), ADA (AAR05843), YU2.DG (M93258), BG1168.1 (AAW64258), 6101 (AAT36747), 7165.18 (AAW64252), 92US715 (AAB04079), AC10 (AAW64261), BL01.DG (AAN39728), PVO.4 (AAW64259), QH0515.01 (AAW64255) and QH0692.42 (AAW64254).
(TIF)

S1 Table Neutralization analysis of vaccine sera from a gp160∆CT DNA prime-gp140F trimer boost study. Twenty rabbits (5 groups of 4) were immunized with various gp160∆CT DNA prime, gp140F trimer boost regimens based on the JR-FL isolate. Each group was distinguished by the nature of the plasmid DNA prime, as depicted. Control group E received no DNA priming. Neutralizing ID50s were measured against tier 1 and tier 2 viruses at several time points during the immunization process: after completing the DNA priming phase and after the first and second protein boosts. Purple and blue labels identify the potent serum from animal 647 that was selected for further investigation as well as the vaccine strain-matched JR-FL parent virus, respectively.
(TIF)

S2 Table Neutralization analysis of immune sera from a DNA prime-gp120 monomer boost study. 10 rabbits were immunized with a simple gp120 DNA prime, gp120 monomer boosts. Neutralization ID50s were measured against tier 1 and tier 2 viruses, using the Monogram PhenoSense 18 hour assay. Purple and blue labels identify the neutralizing serum from animal 2922 that was selected for further investigation and the vaccine strain-matched JR-FL tier 2 parent virus, respectively.
(TIF)

S3 Table Vaccine serum tier 1 virus neutralization. Serum neutralization was measured against the globally sensitive JR-FL A328G mutant (tier 1A phenotype) and SF162 (tier 1B phenotype), by the TZM-bl assay. The neutralization sensitivity profile of the A328G mutant was reported recently in detail (ref [28]).
(TIF)

S4 Table Rabbit sera do not target MPER or common bnAb residue contacts. Selected sera were measured for neutralization activity in a post-CD4.CCR5 assay in which only MPER nAbs can neutralize. Their activities were also measured against mutants N160A, N295Q and N332Q, which are contacts of known bnAbs. Plasma BB34 was included as a reference control.
(TIF)

S5 Table Sensitivity of a multi-clade panel of pseudoviruses to potent rabbit sera and mAbs. N197 glycan-removing mutants of a multi-clade panel of pseudoviruses including clades, A, B, C and B' were generated. Each mutant and its parent were then assessed for their sensitivities to rabbit vaccine sera (ID50s) and a panel of mAbs (IC50s in μg/ml). To calculate mean IC50s, measurements outside of the range of the assay (indicated by >50) were assigned as 50.
(TIF)
