# Supplementary figures and images for: Vaccine-Elicited Tier 2 HIV-1 Neutralizing Antibodies Bind to Quaternary Epitopes Involving Glycan-Deficient Patches Proximal to the CD4 Binding Site
Source: PLoS Pathog. 2015 May 29;11(5):e1004932. doi: 10.1371/journal.ppat.1004932 (PMC4449185; doi:10.1371/journal.ppat.1004932)

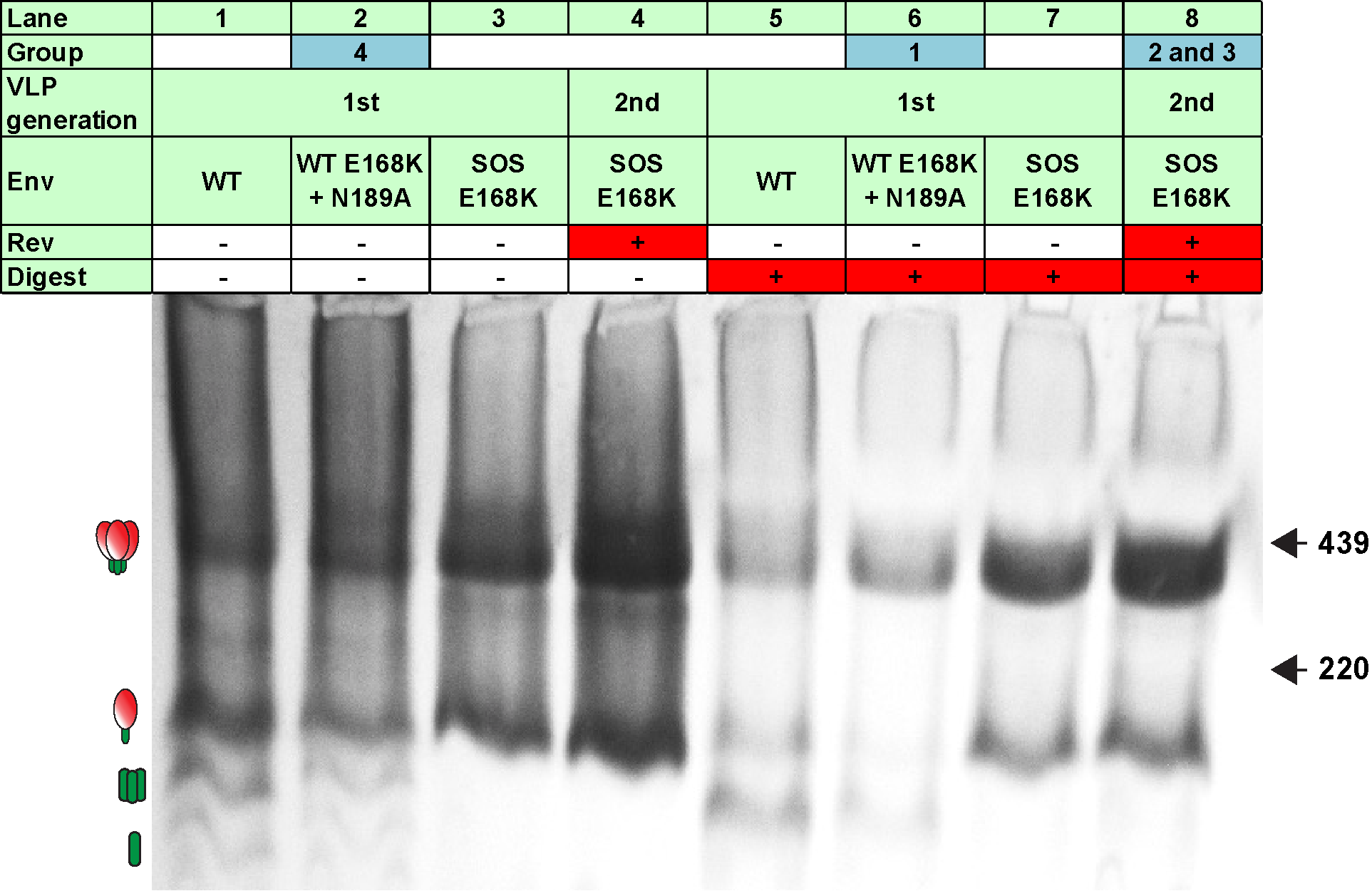

Supplement: S1 Fig — Env liberated from various VLPs was investigated by BN-PAGE-Western blot, probing with a cocktail of α-gp120 and α-gp41 mAbs. Lanes corresponding to the immunogens using in animal groups 1–4 are indicated. Ferritin was used as a molecular weight marker. Major bands are identified by cartoons representing (from top to bottom) native trimers, monomeric gp160, trimeric and monomeric gp41 stumps. (TIF) [file ppat.1004932.s001.tif]

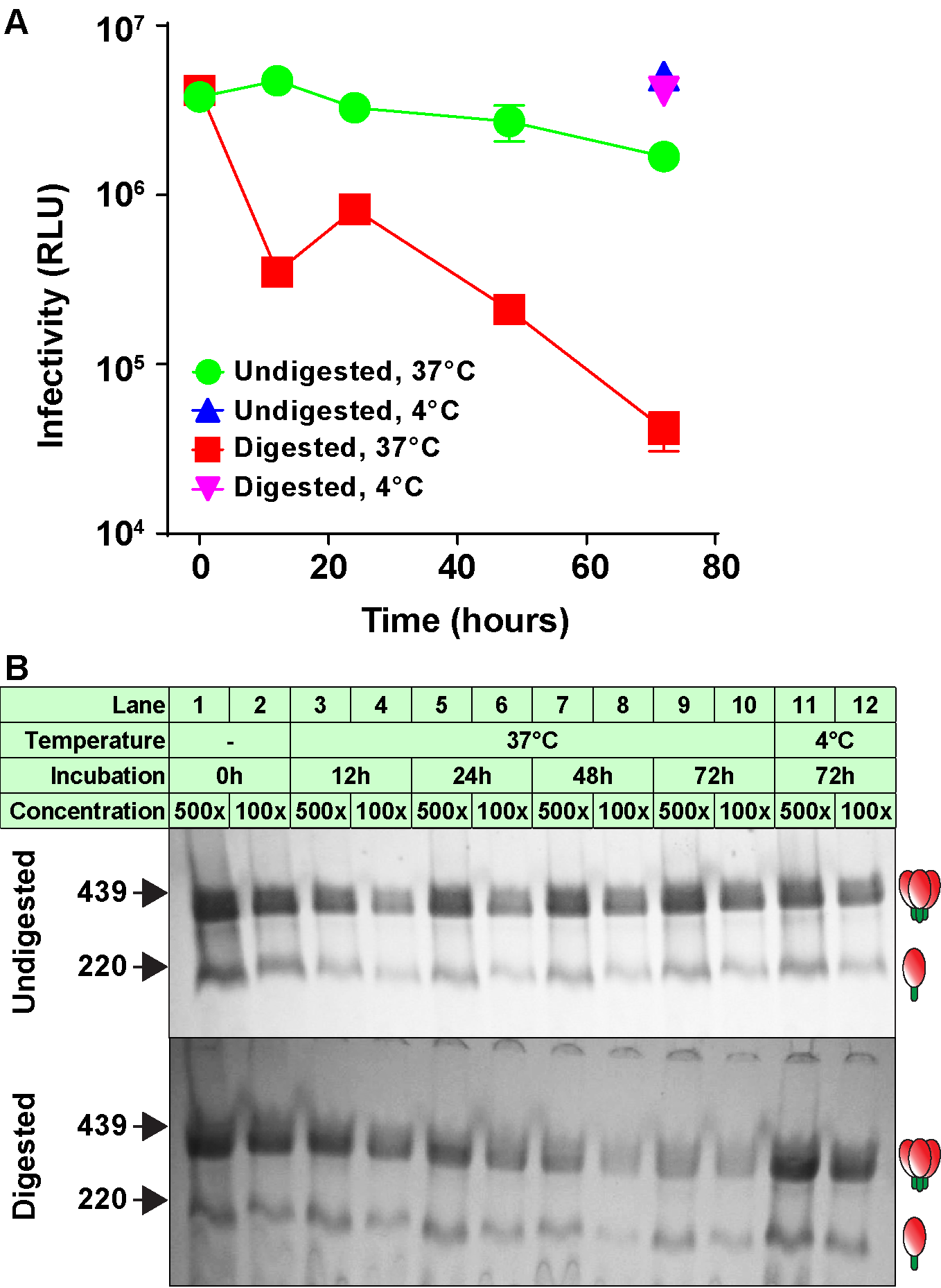

Supplement: S2 Fig — A) Infectivity decay of digested or undigested 1st generation WT E168K+N189A VLPs was measured over time at 4°C and 37°C. B) BN-PAGE-Western blot analysis of the decay of undigested (upper panel) and digested (lower panel) 2nd generation SOS E168K trimer VLPs at 37°C over time. Major bands are identified by cartoons that represent the native trimer and monomeric UNC gp160. (TIF) [file ppat.1004932.s002.tif]

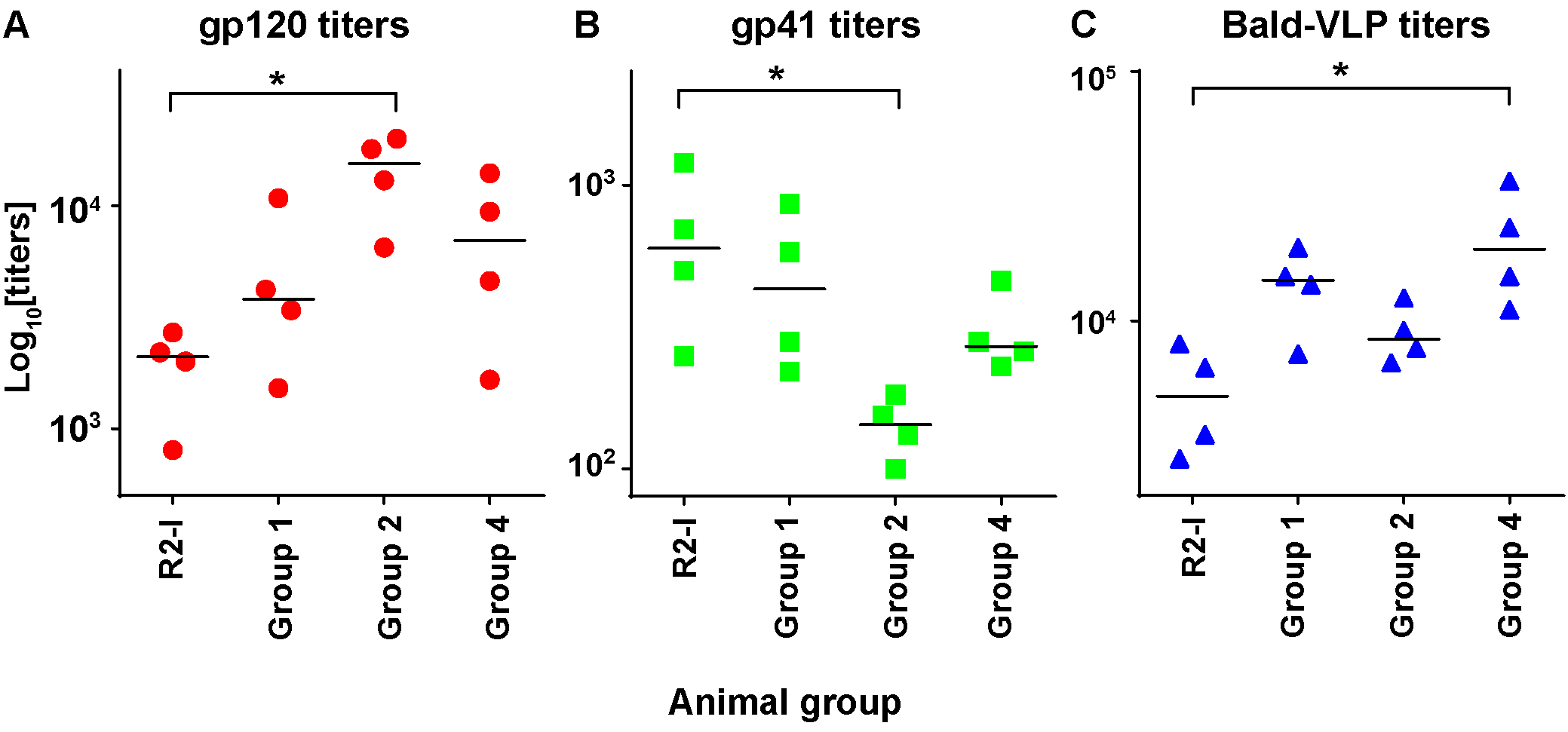

Supplement: S3 Fig — A) Monomeric gp120, B) gp41 and C) bald VLP binding titers were compared between the current groups of rabbits (1, 2 and 4) and those of our previous study (Group R2-I in Fig 5 of ref. [28]). Mean titers are indicated by horizontal lines. Asterisks indicate significant differences (p<0.05) by Mann-Whitney two-tailed test. (TIF) [file ppat.1004932.s003.tif]

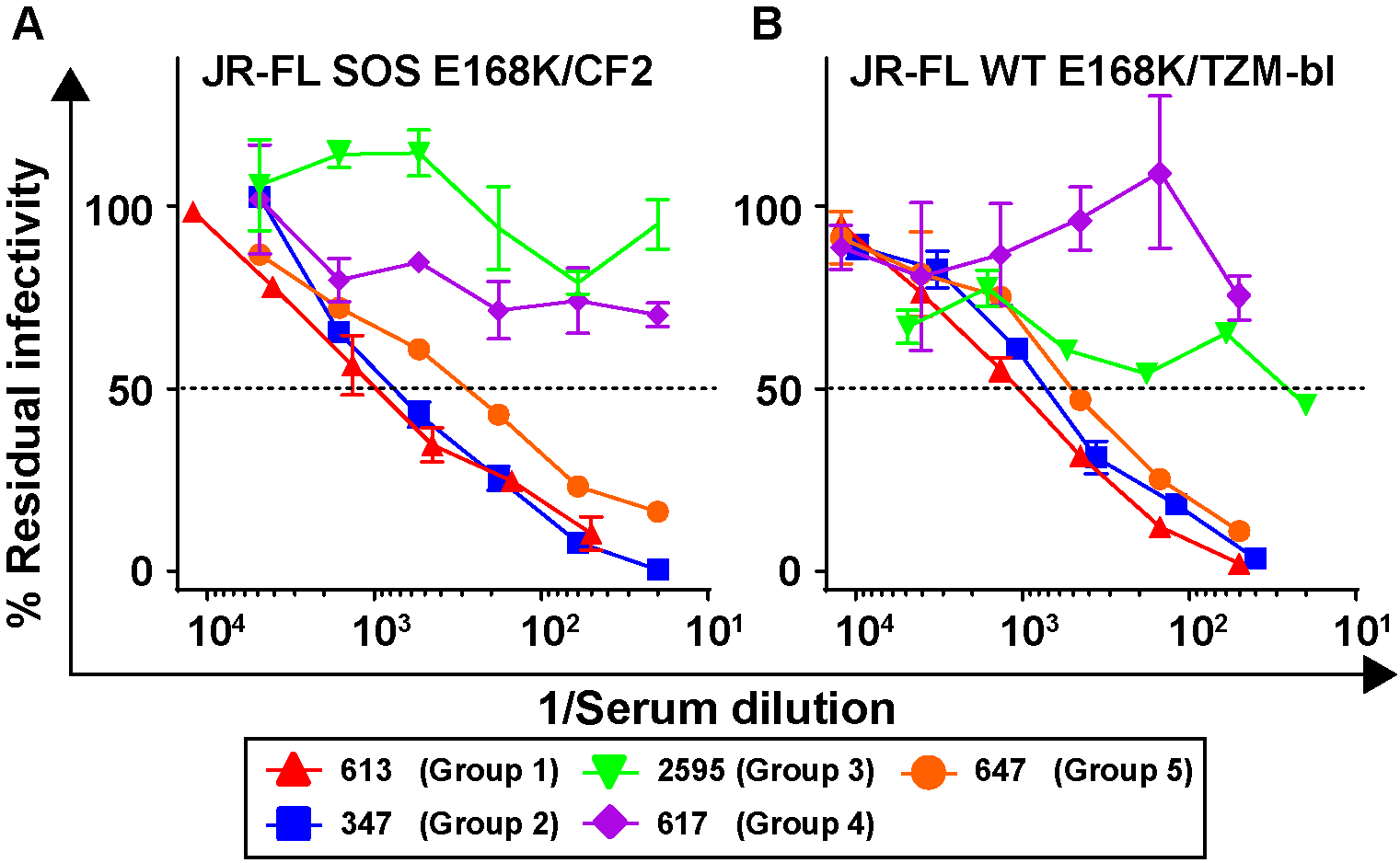

Supplement: S4 Fig — Representative sera from each animal group were titrated against A) JR-FL SOS E168K pseudovirus in the CF2 assay and B) JR-FL WT E168K pseudovirus in the TZM-bl assay. (TIF) [file ppat.1004932.s004.tif]

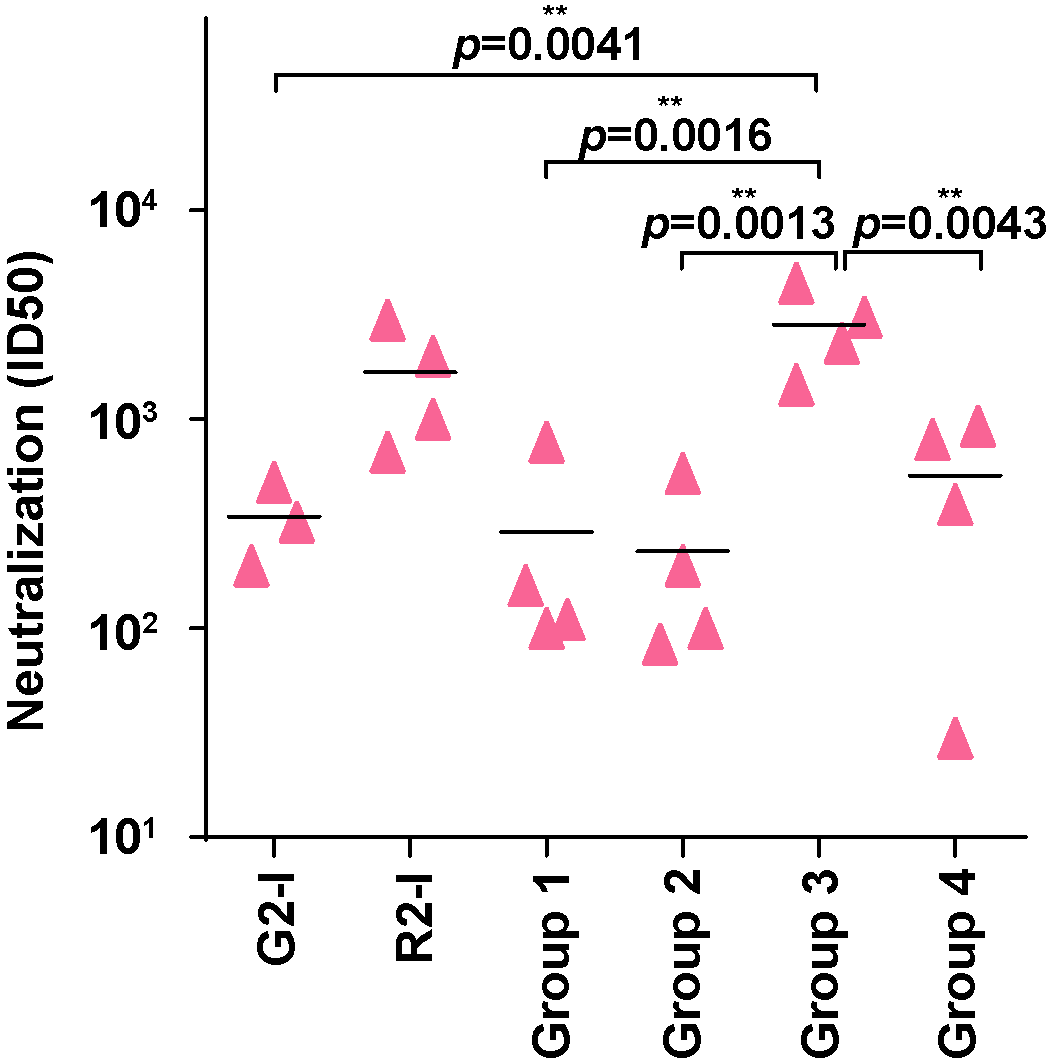

Supplement: S5 Fig — Mean JR-FL A328G tier 1 nAb titers were compared in all 4 current groups of VLP sera and in groups of guinea pig (G2-I) and rabbit (R2-I) sera from our previous study [28]. Statistical significance is indicated with p value arising from an ANOVA one-way comparison between all groups. (TIF) [file ppat.1004932.s005.tif]

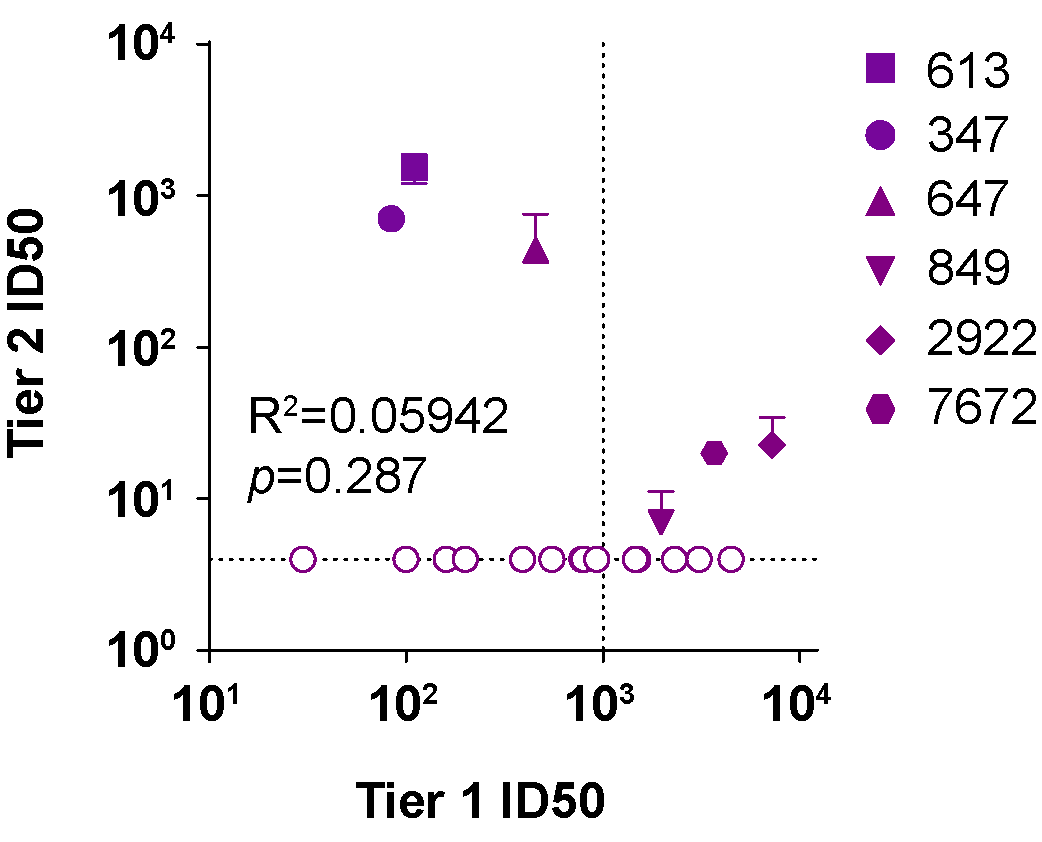

Supplement: S6 Fig — Vaccine serum nAb titers against the tier 2 parent JR-FL E168K virus and tier 1 JR-FL A328G virus measured in CF2 cells were compared in a scatterplot. Filled symbols depict those with detectable tier 2 nAbs. Open symbols are from sera that lacked detectable tier 2 nAbs, which are arbitrarily assigned with titers of 1:4. An r2 and p value was calculated for all the data using linear regression best-fit line. (TIF) [file ppat.1004932.s006.tif]

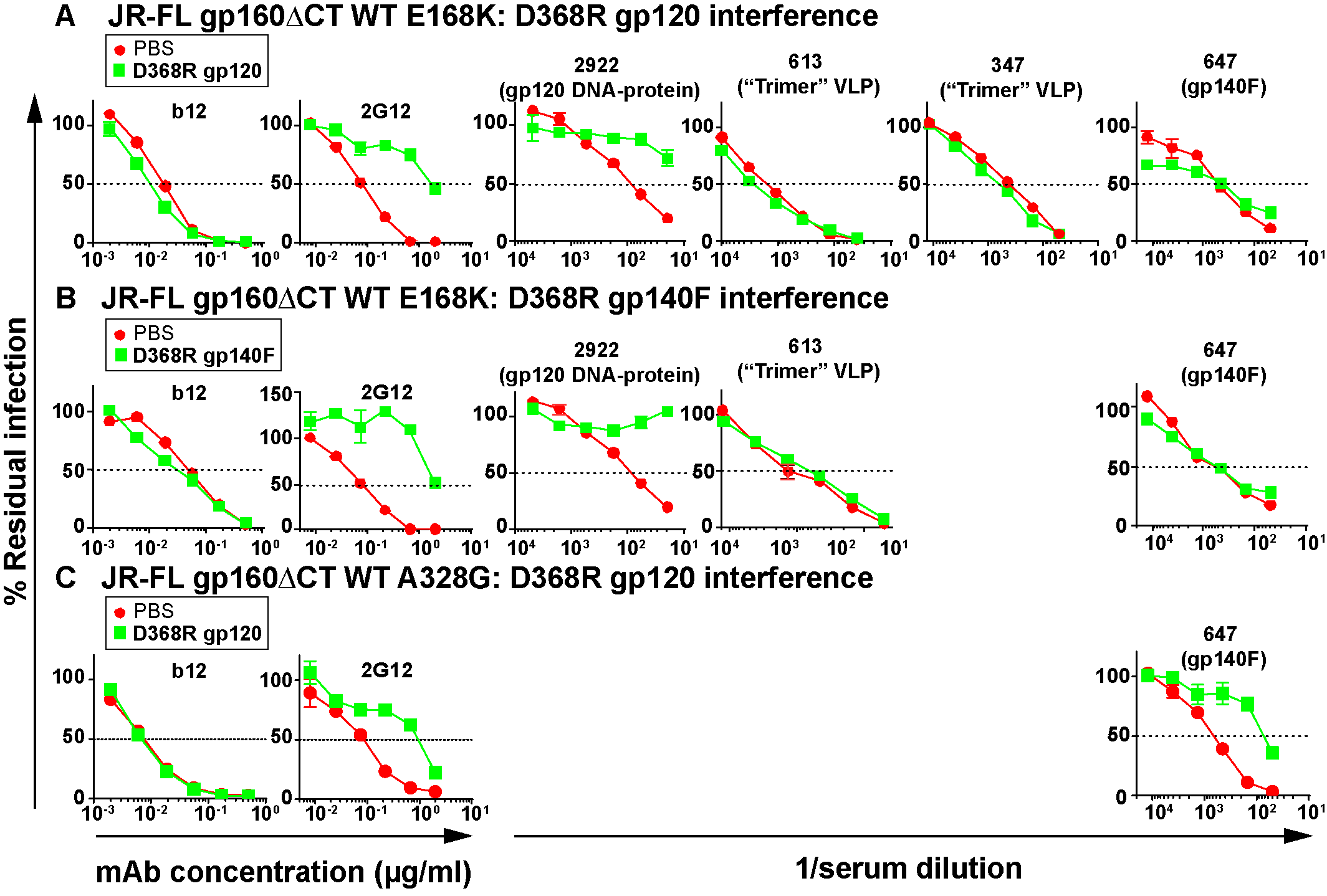

Supplement: S7 Fig — Effect of adding 10μg/ml of purified D368R mutant versions of JR-FL monomeric gp120 (parts A and C) and gp140F trimer (part B) on serum and mAb neutralization of JR-FL gp160∆CT WT E168K (parts A and B) and JR-FL gp160∆CT WT A328G (part C) in the TZM-bl assay. (TIF) [file ppat.1004932.s007.tif]

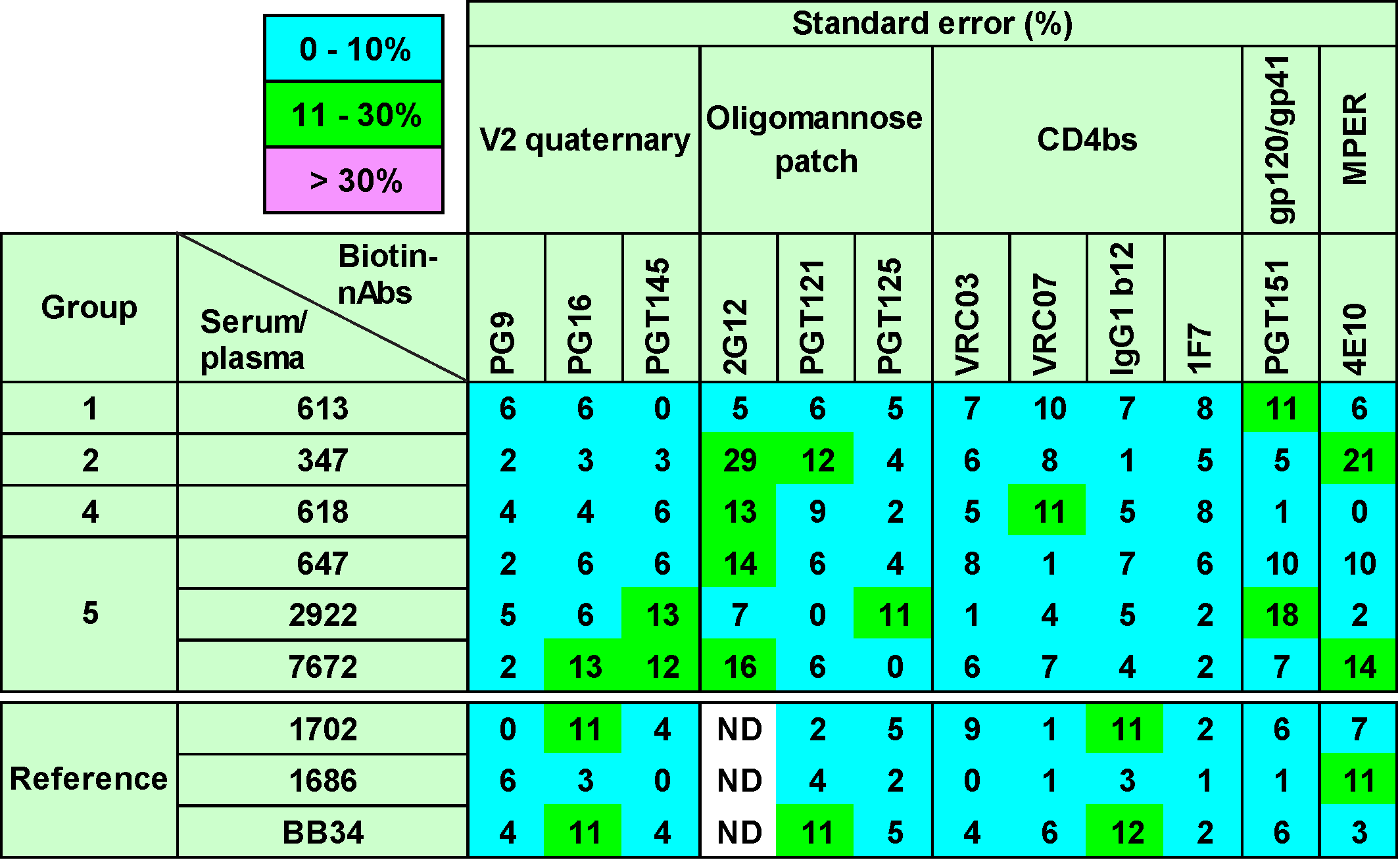

Supplement: S8 Fig — This data partners with competition data in Fig 5. (TIF) [file ppat.1004932.s008.tif]

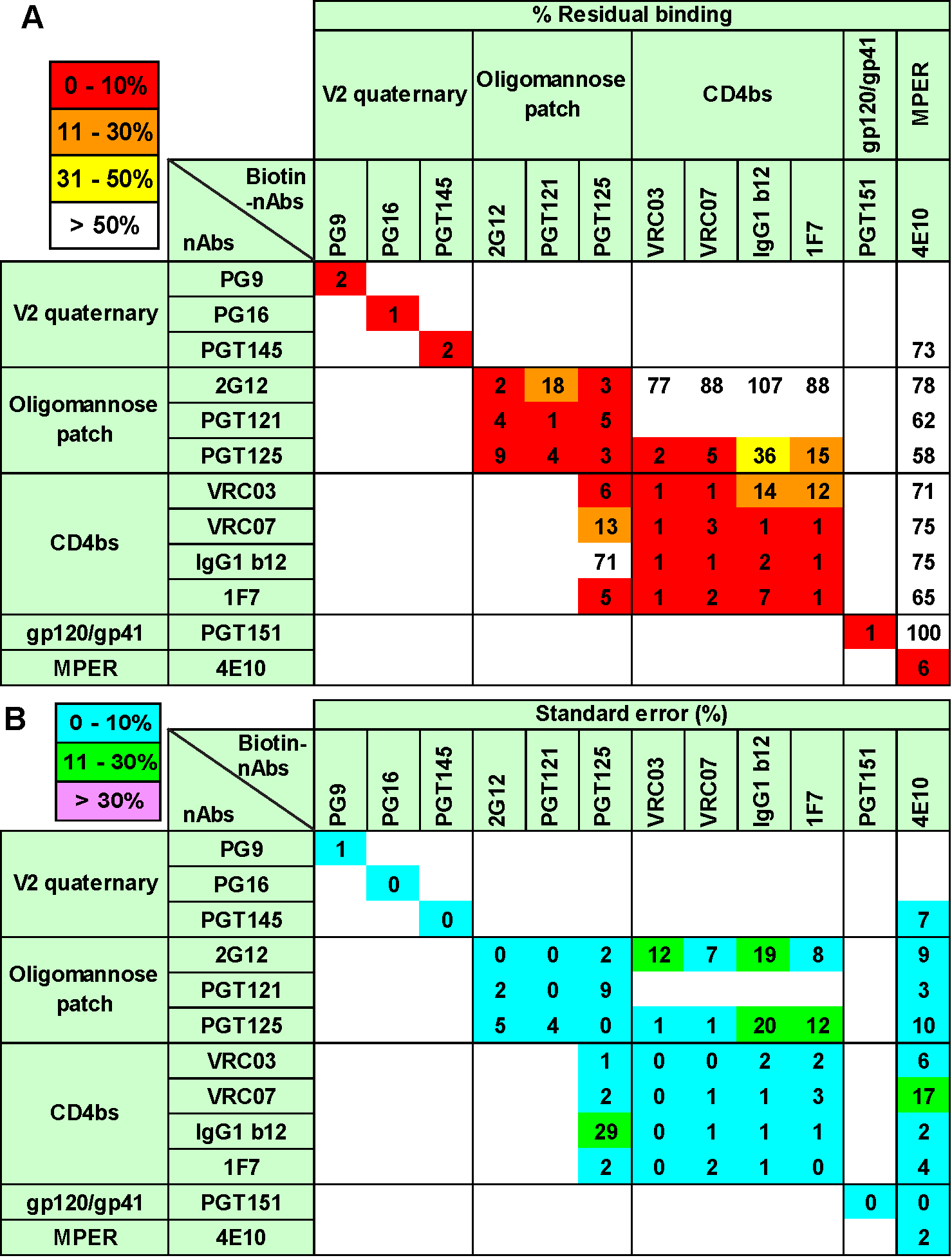

Supplement: S9 Fig — A) As in Fig 5, competition data are shown as percentages; B) the standard errors of data in part A) are shown. (TIF) [file ppat.1004932.s009.tif]

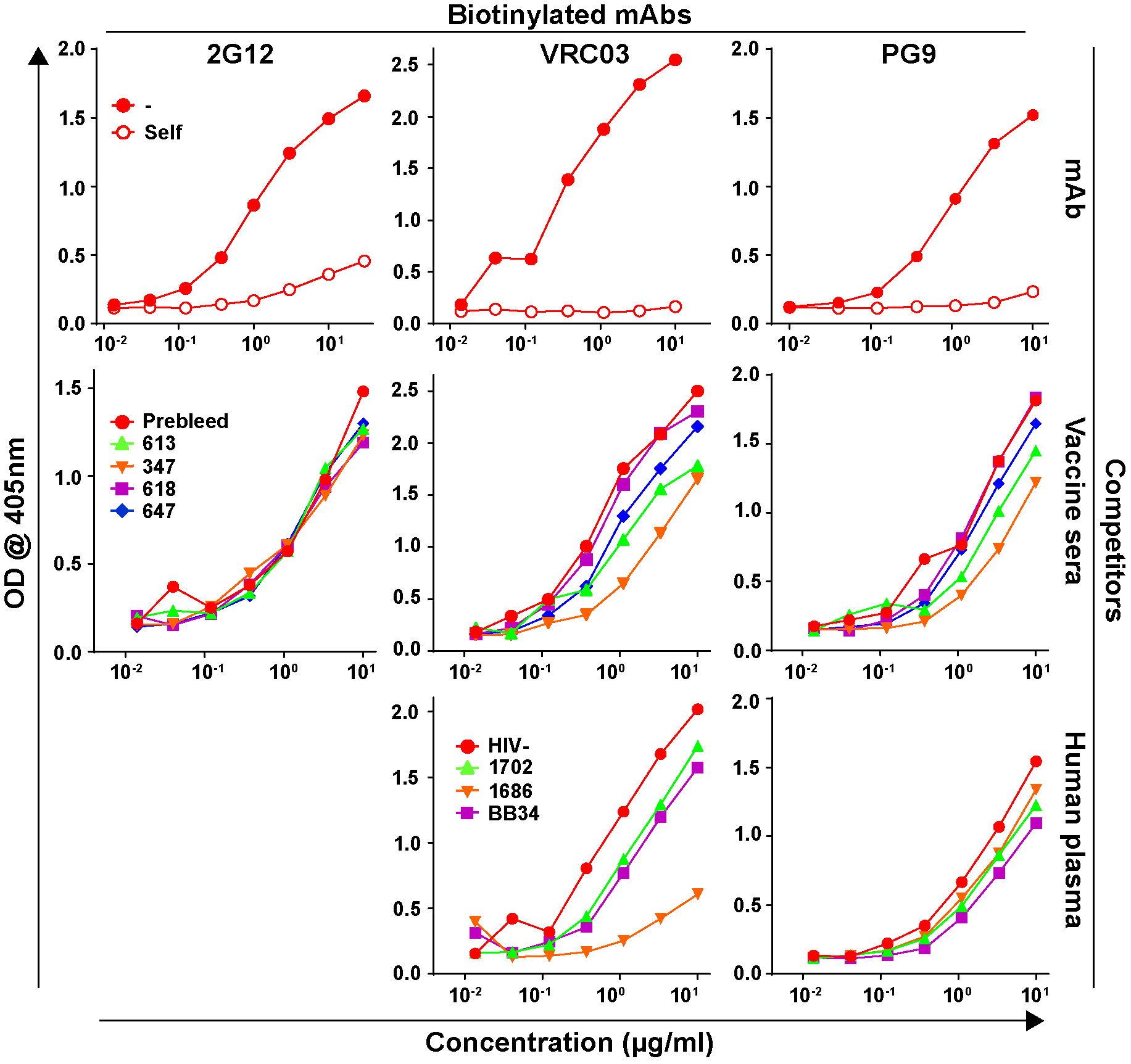

Supplement: S10 Fig — Here we show the effects of competitor antibodies on the binding of biotinylated mAbs over a range of concentrations. (TIF) [file ppat.1004932.s010.tif]

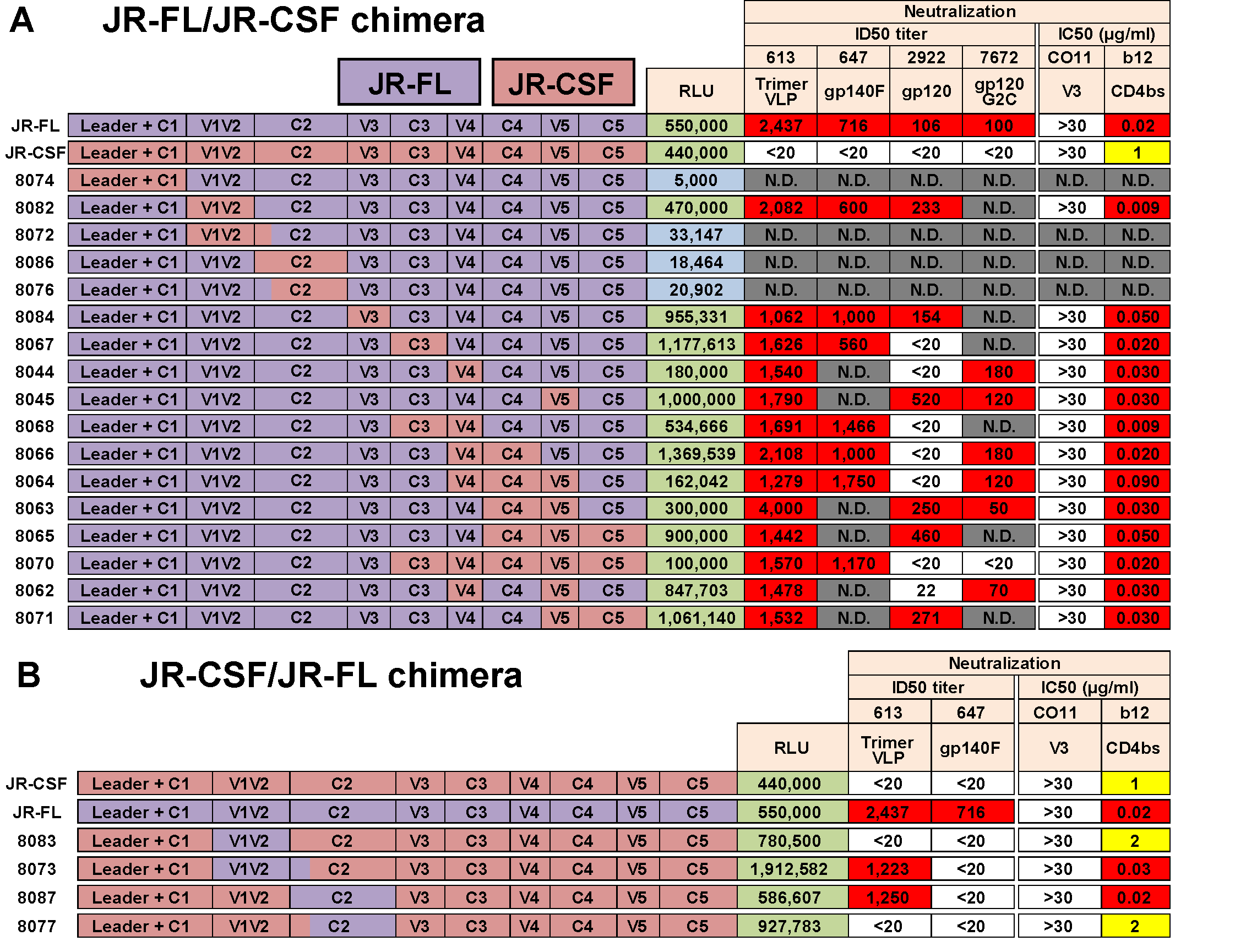

Supplement: S11 Fig — Chimeras comprising of a A) JR-FL Env or B) JR-CSF Env background with JR-FL/JR-CSF domain swaps, color coded as indicated, were evaluated for their infectivity (in relative light units; RLU) and sensitivity to vaccine sera and mAb CO11 and b12 neutralization. (TIF) [file ppat.1004932.s011.tif]

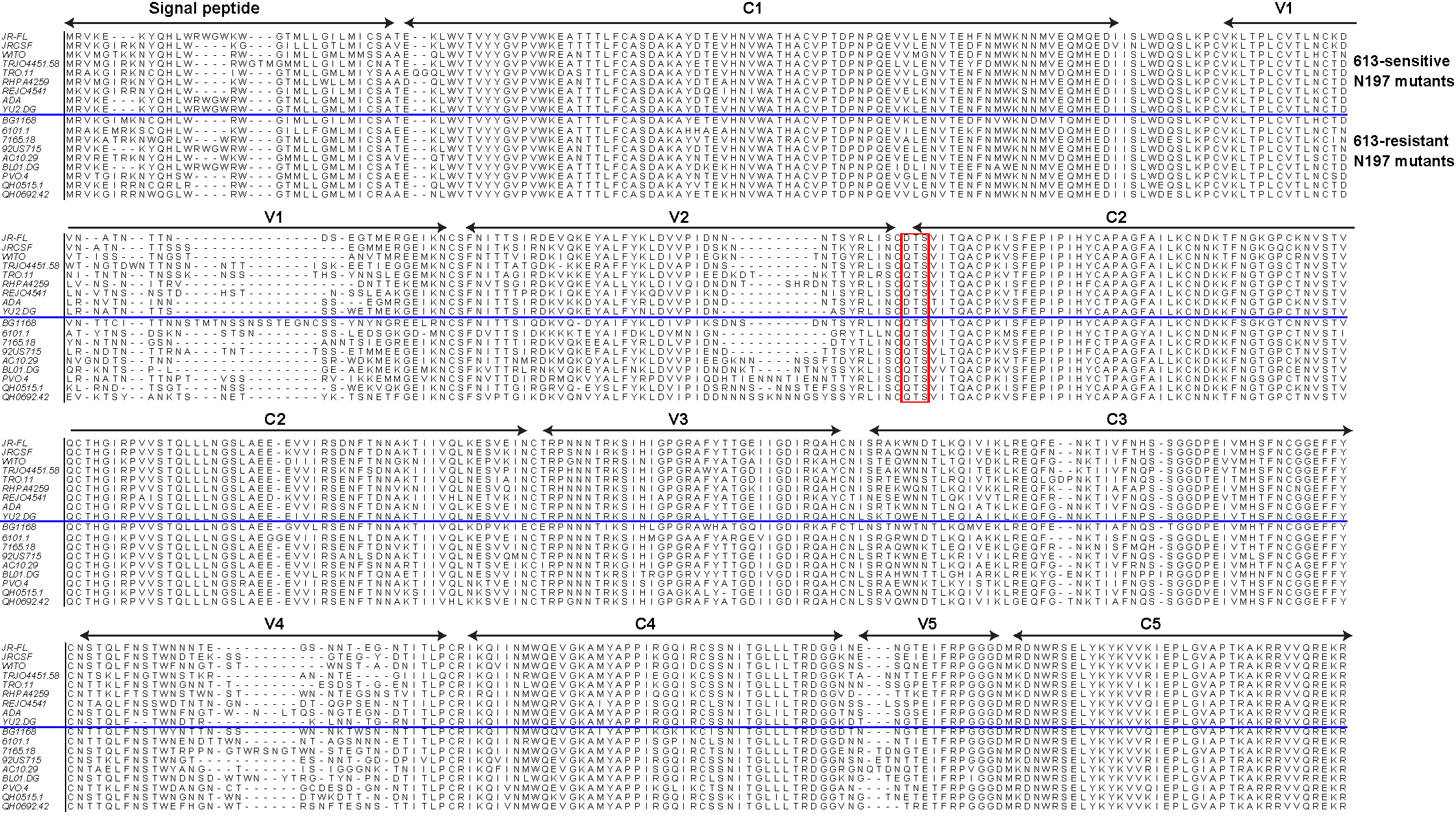

Supplement: S12 Fig — Amino acid sequences of clade B N197 mutant viruses from S5 Table were aligned by Tcoffee method using JalView software [90]. Only Envs from clade B were aligned and partitioned into two sections: the top being 613 serum-sensitive, the bottom being 613 serum-resistant. N197 glycan position is highlighted with red box. The parent clade B Env protein Genbank accession number are as follows: JR-FL (AAB05604), JR-CSF (AAB03749), WITO (AAW64266), TRJO4551 (AAW64265), TRO.11 (AAW64260), RHPA.4259 (AAW64262), REJO4541 (AAW64264), ADA (AAR05843), YU2.DG (M93258), BG1168.1 (AAW64258), 6101 (AAT36747), 7165.18 (AAW64252), 92US715 (AAB04079), AC10 (AAW64261), BL01.DG (AAN39728), PVO.4 (AAW64259), QH0515.01 (AAW64255) and QH0692.42 (AAW64254). (TIF) [file ppat.1004932.s012.tif]

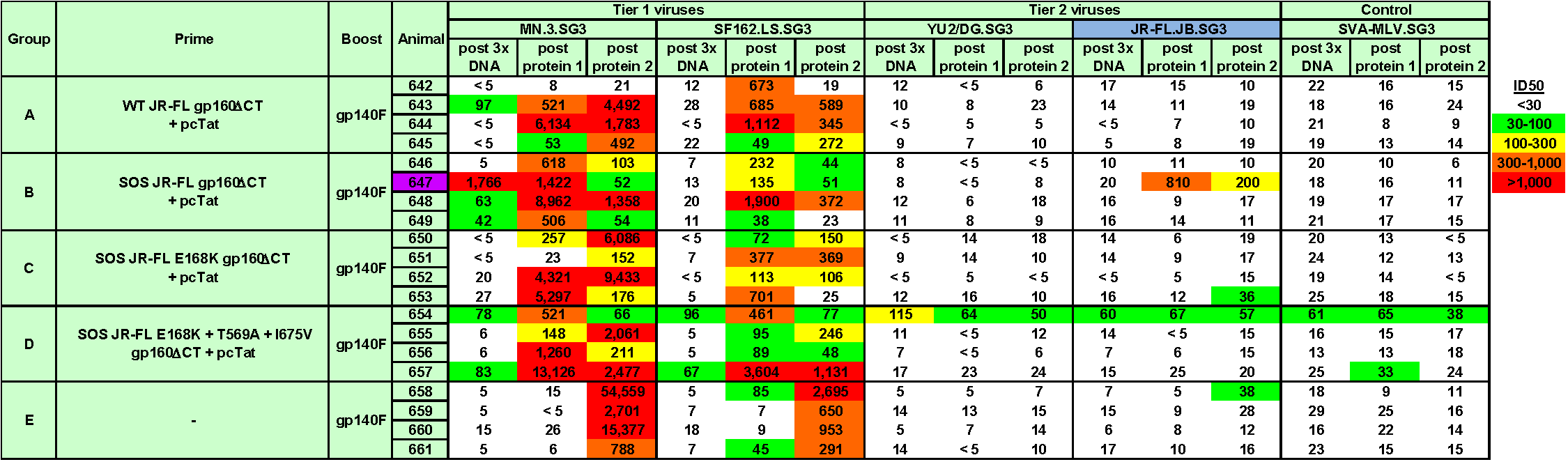

Supplement: S1 Table — Twenty rabbits (5 groups of 4) were immunized with various gp160∆CT DNA prime, gp140F trimer boost regimens based on the JR-FL isolate. Each group was distinguished by the nature of the plasmid DNA prime, as depicted. Control group E received no DNA priming. Neutralizing ID50s were measured against tier 1 and tier 2 viruses at several time points during the immunization process: after completing the DNA priming phase and after the first and second protein boosts. Purple and blue labels identify the potent serum from animal 647 that was selected for further investigation as well as the vaccine strain-matched JR-FL parent virus, respectively. (TIF) [file ppat.1004932.s013.tif]

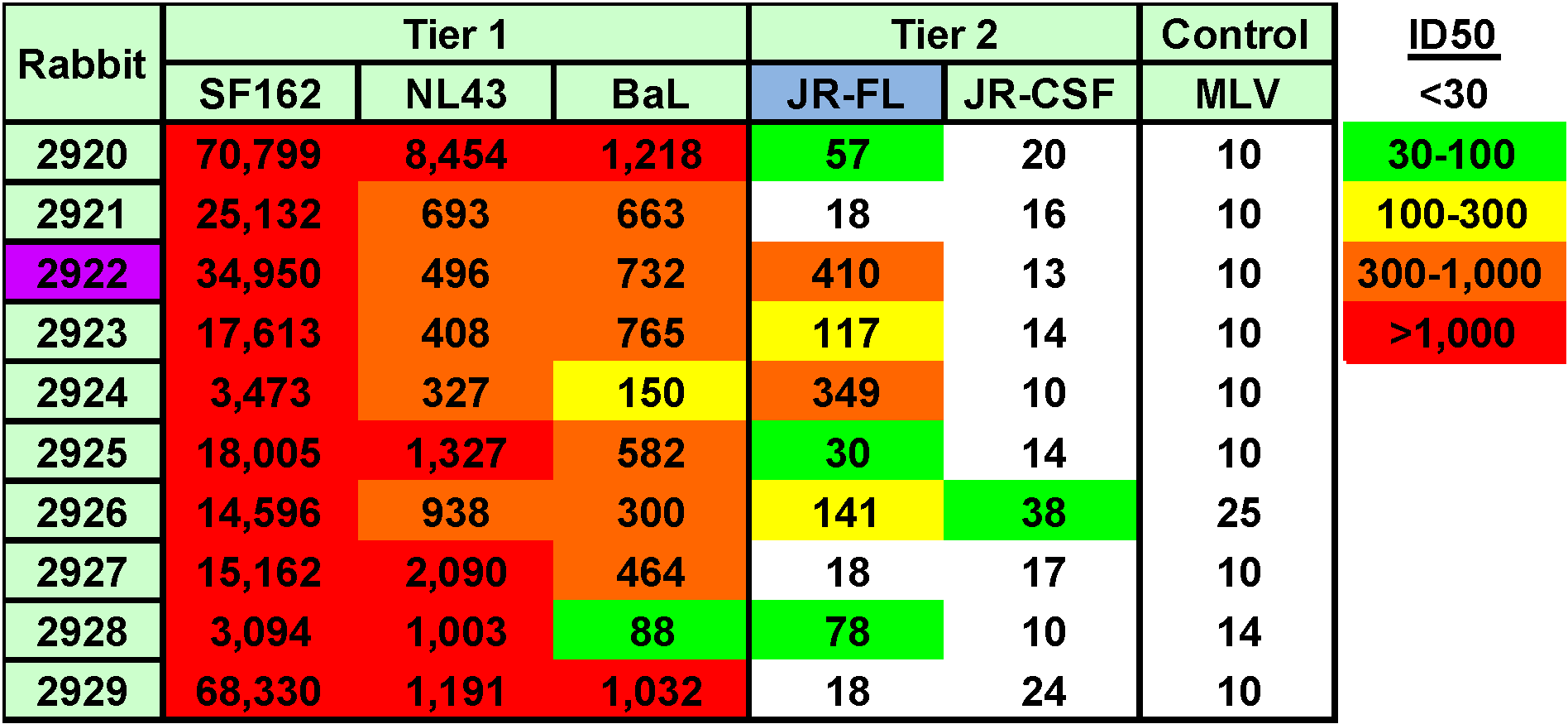

Supplement: S2 Table — 10 rabbits were immunized with a simple gp120 DNA prime, gp120 monomer boosts. Neutralization ID50s were measured against tier 1 and tier 2 viruses, using the Monogram PhenoSense 18 hour assay. Purple and blue labels identify the neutralizing serum from animal 2922 that was selected for further investigation and the vaccine strain-matched JR-FL tier 2 parent virus, respectively. (TIF) [file ppat.1004932.s014.tif]

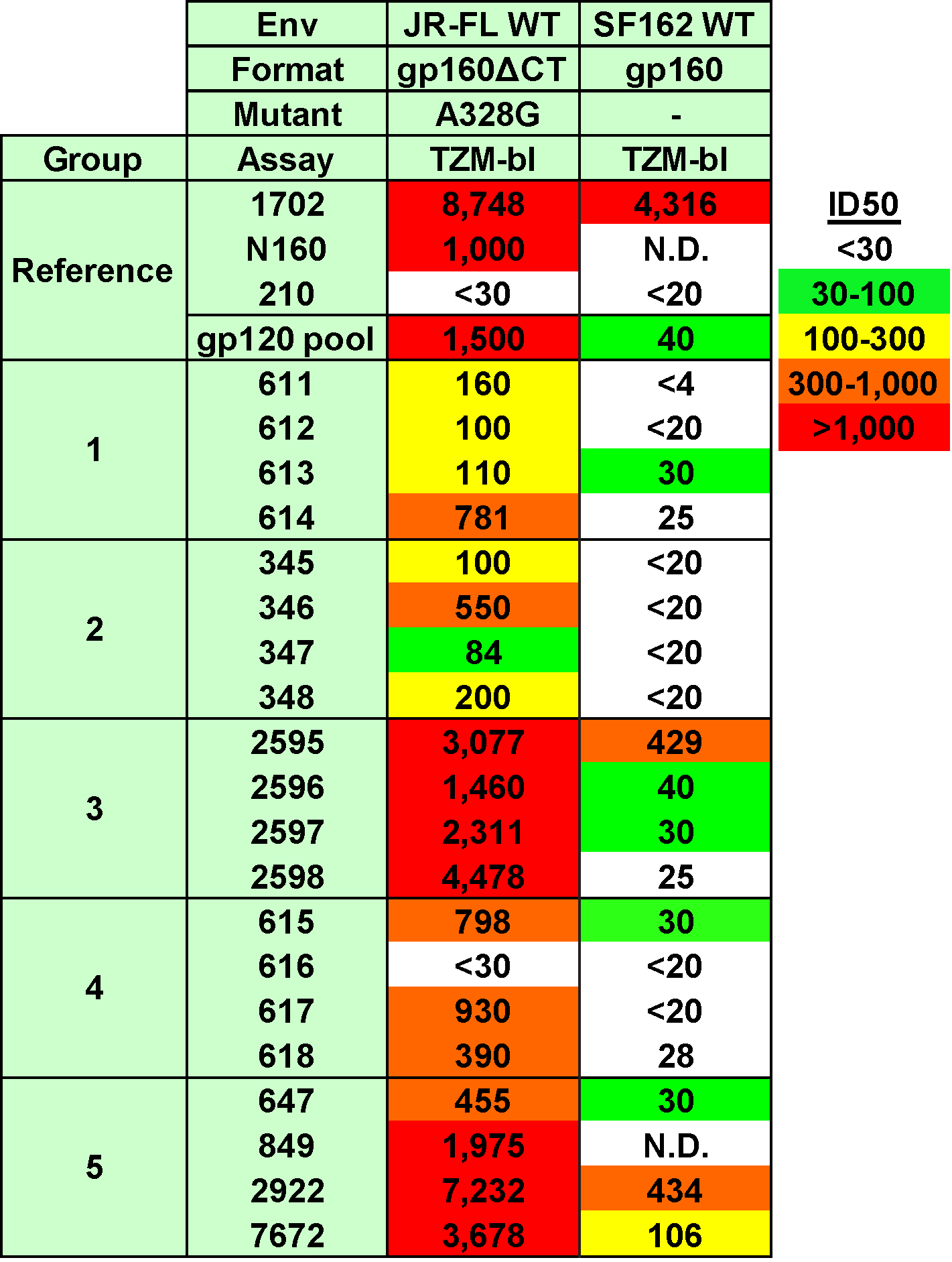

Supplement: S3 Table — Serum neutralization was measured against the globally sensitive JR-FL A328G mutant (tier 1A phenotype) and SF162 (tier 1B phenotype), by the TZM-bl assay. The neutralization sensitivity profile of the A328G mutant was reported recently in detail (ref [28]). (TIF) [file ppat.1004932.s015.tif]

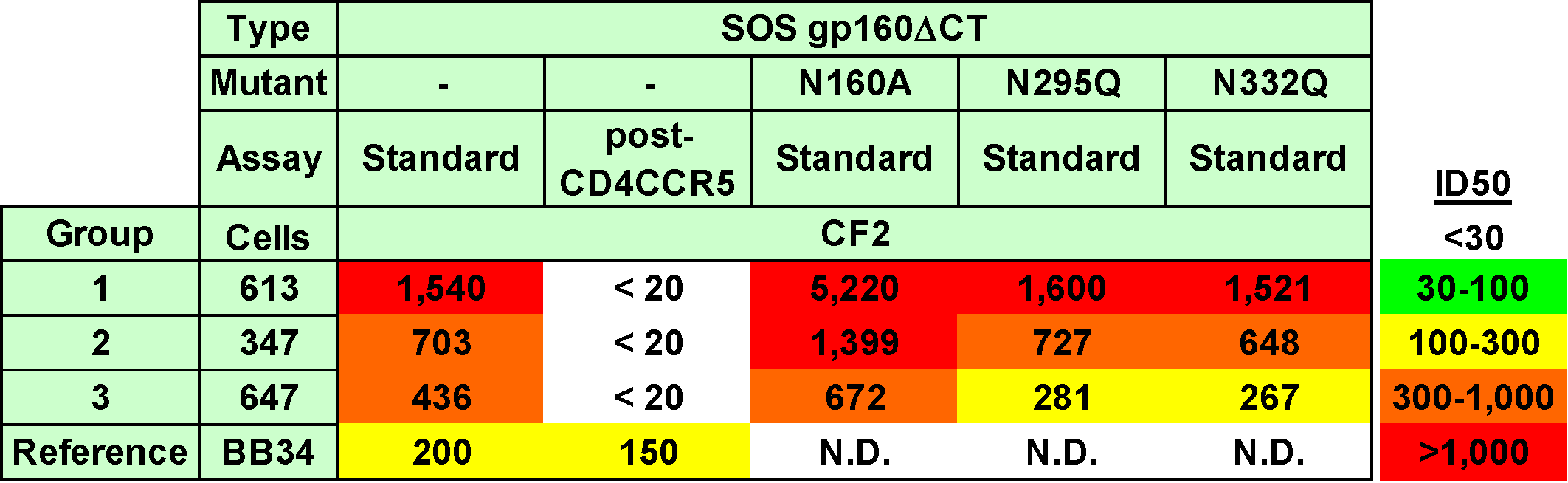

Supplement: S4 Table — Selected sera were measured for neutralization activity in a post-CD4.CCR5 assay in which only MPER nAbs can neutralize. Their activities were also measured against mutants N160A, N295Q and N332Q, which are contacts of known bnAbs. Plasma BB34 was included as a reference control. (TIF) [file ppat.1004932.s016.tif]

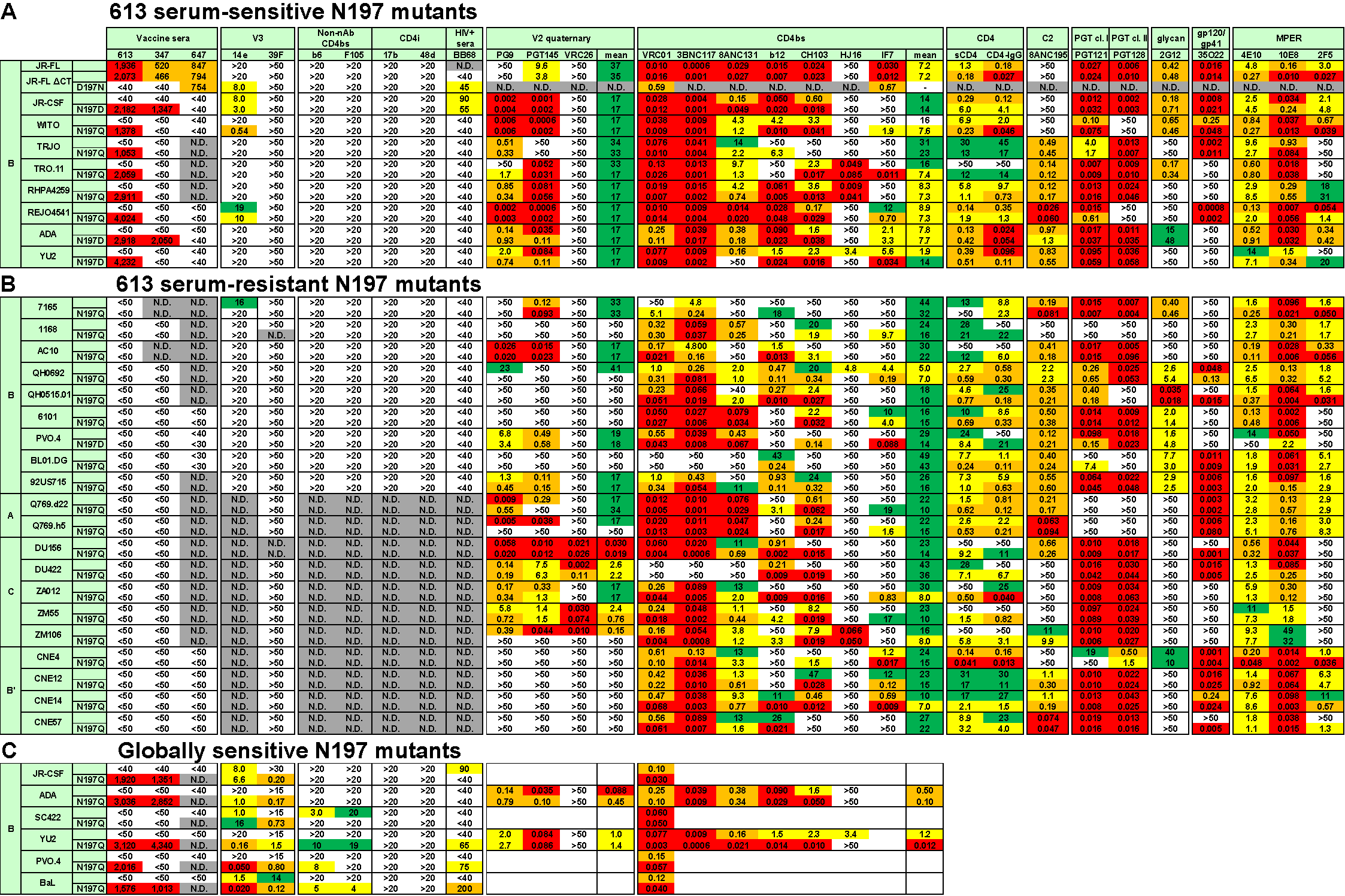

Supplement: S5 Table — N197 glycan-removing mutants of a multi-clade panel of pseudoviruses including clades, A, B, C and B' were generated. Each mutant and its parent were then assessed for their sensitivities to rabbit vaccine sera (ID50s) and a panel of mAbs (IC50s in μg/ml). To calculate mean IC50s, measurements outside of the range of the assay (indicated by >50) were assigned as 50. (TIF) [file ppat.1004932.s017.tif]
